# A comprehensive analysis of 195 DNA methylomes reveals shared and cell-specific features of partially methylated domains

**DOI:** 10.1186/s13059-018-1510-5

**Published:** 2018-09-28

**Authors:** Abdulrahman Salhab, Karl Nordström, Gilles Gasparoni, Kathrin Kattler, Peter Ebert, Fidel Ramirez, Laura Arrigoni, Fabian Müller, Julia K. Polansky, Cristina Cadenas, Jan G.Hengstler, Thomas Lengauer, Thomas Manke, DEEP Consortium, Jörn Walter

**Affiliations:** 1 Department of Genetics, Saarland University, Campus Saarbrücken, Saarbrücken, 66123 Germany; 2 Department of Computational Biology and Applied Algorithmics, Max Planck Institute for Informatics, Saarbrücken, 66123 Germany; 3 Max Planck Institute of Immunobiology and Epigenetics, Freiburg, 79108 Germany; 4 Berlin-Brandenburg Center for Regenerative Therapies at the Charité, Berlin, Germany; 5 University Medicine Berlin and German Rheumatism Research Centre, Berlin, Germany; 6 Leibniz Research Center for working Environment and Human Factors IfADo, Dortmund, 44139 Germany

**Keywords:** Partially methylated domains, Heterochromatin, Replication timing, Proliferation

## Abstract

**Background:**

Partially methylated domains are extended regions in the genome exhibiting a reduced average DNA methylation level. They cover gene-poor and transcriptionally inactive regions and tend to be heterochromatic. We present a comprehensive comparative analysis of partially methylated domains in human and mouse cells, to identify structural and functional features associated with them.

**Results:**

Partially methylated domains are present in up to 75% of the genome in human and mouse cells irrespective of their tissue or cell origin. Each cell type has a distinct set of partially methylated domains, and genes expressed in such domains show a strong cell type effect. The methylation level varies between cell types with a more pronounced effect in differentiating and replicating cells. The lowest level of methylation is observed in highly proliferating and immortal cancer cell lines. A decrease of DNA methylation within partially methylated domains tends to be linked to an increase in heterochromatic histone marks and a decrease of gene expression. Characteristic combinations of heterochromatic signatures in partially methylated domains are linked to domains of early and middle S-phase and late S-G2 phases of DNA replication.

**Conclusions:**

Partially methylated domains are prominent signatures of long-range epigenomic organization. Integrative analysis identifies them as important general, lineage- and cell type-specific topological features. Changes in partially methylated domains are hallmarks of cell differentiation, with decreased methylation levels and increased heterochromatic marks being linked to enhanced cell proliferation. In combination with broad histone marks, partially methylated domains demarcate distinct domains of late DNA replication.

**Electronic supplementary material:**

The online version of this article (10.1186/s13059-018-1510-5) contains supplementary material, which is available to authorized users.

## Background

DNA methylation is an epigenetic hallmark with an important role in gene and genome regulation. Changes in the genome-wide landscape of DNA methylation are extensively studied in the context of small regulatory regions like CpG islands [1], CpG shores [2], and proximal and distal regulatory regions [3]. With the first genome-wide bisulfite-based DNA methylation analyses, a new term, partially methylated domains (PMDs), was introduced by Lister et al. [4] referring to long genomic regions in the range of hundreds of kilo-basepairs (kb) characterized by highly disordered methylation levels. They were initially discovered in the fibroblast cell line IMR90 but cannot be observed in human embryonic stem cells H1.

It has been shown later that PMDs are enriched with heterochromatic histone modifications such as H3K27me3 and that they are gene-poor and less active [5, 6] than other genomic regions. Several studies have since reported PMDs in various cell types: medulloblastoma [6], adipocyte tissue [7], SH-SY5Y neuronal cells [8], and human cancers [5, 9–11]. PMDs in cancer cells are linked to late replication and nuclear lamina-associated regions [10]. The first non-cancer primary human tissue type with PMDs has been reported in placenta [12] and were defined using a hidden Markov model (HMM) rather than applying a threshold of methylation level. Recently, we, as part of the DEEP consortium http://www.deutsches-epigenom-programm.de/, published the first primary human cells, CD4+ T cells, with PMDs [13]. We showed that progressive loss of DNA methylation correlates with T cell memory differentiation and happens predominantly in PMDs. Burger et al. [3] implemented an HMM-based detection method called MethylSeekR to define PMDs and separate them from highly methylated domains (HMDs) and short (regulatory) regions that come in two types (methylation below 50%): lowly methylated regions (LMRs, CpG poor regions, less than 30 CpGs) and unmethylated regions (UMRs, mostly CpG islands, more than 30 CpGs). LMRs and UMRs are relatively short (a few hundred to a few thousand basepairs) and correspond to distal and proximal regulatory elements, respectively [14]. Tools such as MethylSeekR are very useful for exploring the methylome landscape on a large scale and help to discriminate the large domains, from the small regulatory regions.

In collaboration with our colleagues in the international human epigenome consortium IHEC http://ihec-epigenomes.org/, we contributed to generating a large epigenome cohort for numerous primary cell types from human and mouse. WGBS data serve as an invaluable resource for studying PMDs in primary cells. PMDs represent a new aspect for studying the DNA methylation landscape on a genome-wide level apart from the context of regulatory regions that have been studied extensively and pose the question whether DNA methylation has an impact on the genome organization. At the same time, it has become quite clear that cells in vitro behave differently from primary cells, for instance regarding methylation levels. Thus, it is important to compare the methylome of primary cells and cell lines in order to validate in vitro systems and afford an appropriate interpretation of the data.

Here, we investigate the genome-wide organization of PMDs across a comprehensive spectrum of available WGBS data generated by IHEC members, DEEP http://www.deutsches-epigenomprogramm.de/, Blueprint www.blueprint-epigenome.eu, and Roadmap http://www.roadmapepigenomics.org/, together with other public data in order to gain insights into PMDs. In addition, we integrated WGBS data with other epigenetic data, ChIP-seq, RNA-seq, Hi-C and Repli-seq, in an attempt to describe the interaction between DNA methylation and chromatin formation in order to understand how they impact cell division, differentiation, and the higher order chromatin structure. Moreover, we propose a new integrative approach to exploring and interpreting methylome topologies using WGBS data, an approach very much needed as the amount of such data is growing rapidly.

## Results

### Partially methylated domains are cell type discriminators

**Fig. 1:**
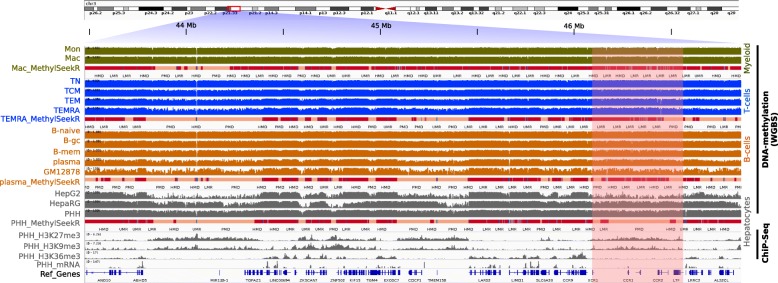
Genome-wide DNA methylation segmentation across different cell types. IGV snapshot showing DNA methylation profiles of different cell types (from top to bottom: monocytes, macrophages, naive/central/effector/terminal memory T cells, naive/germinal center/class switched memory B cells, plasma, GM12878, HepG2, HepaRG, and primary human hepatocytes) with the corresponding MethylSeekR segments: highly methylated domains (red); HMDs, partially methylated domains (light pink); PMDs, low methylated regions (light blue); LMRs and unmethylated regions (blue); UMRs (only one track per cell type shown for simplicity). Below the block of methylation data, three broad histone marks and RNA-seq profiles of hepatocytes are displayed. PMDs can be seen as long regions with highly disordered methylation levels and tend to be largely overlapping between the different cell types. However, there are cell type-specific PMDs (the highlighted region shows a hepatocyte-specific PMD). PMDs are gene-poor and transcriptionally inactive regions and have heterochromatic signature (H3K27me3 and H3K9me3). In contrast, HMDs are transcriptionally active and rich-gene regions with enrichment of active histone mark H3K36me3

**Fig. 2:**
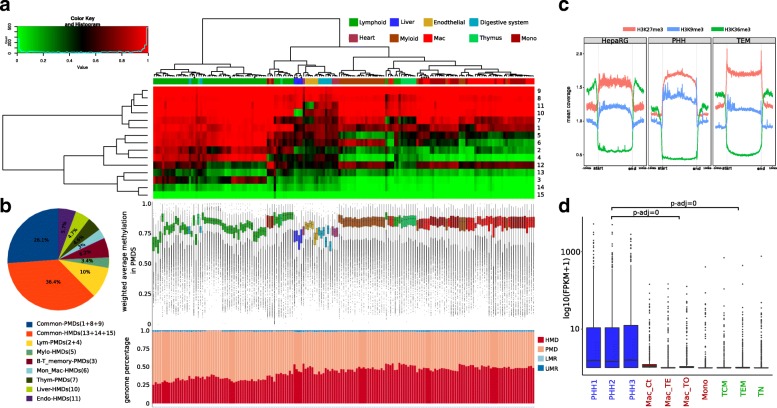
PMD signatures discriminate cell type and global transcriptional control. **a** Colored representation of the emission probabilities calculated by ChromH3M. Samples and states were hierarchically clustered forming six main groups: myeloid, lymphoid, endothelial, liver, digestive system, and heart. Beneath each sample, the corresponding average methylation levels within PMDs is shown as whisker box plots and the percentage of MethylSeekR segments as stacked bar plots. Samples derived from the same cell type clustered together, although they differ in mean methylation level, suggesting that they have more similar PMD structure than the other cell types. PMDs comprise about 50–75% of the genome. **b** Graphical representation of the relative (percentage) contribution of each state in the 15-state ChromH3M model. Twenty-six percent of the genome shares the same PMDs across all samples and roughly one third differ between them. **c** Genome-wide normalized histone mark signals within PMDs (including 100 kb flanking regions). Note the enrichment of heterochromatic marks H3K27me3 and H3K9me3 across PMDs and a depletion of the transcription-coupled mark H3K36me3. **d** Log10-scaled FPKM values of state 10 (Liver-HMDs) associated protein-coding genes. Genes are significantly more highly expressed in hepatocyte samples (PHH) than in macrophages, monocytes, and T cells, according to two-way ANOVA and Tukey HSD post hoc test (see details in the “Methods” section). Only samples marked with star in **a**are used for simplicity and since they belong to one consortium

We collected and surveyed 171 public human WGBS datasets of different primary cell types (hepatocytes, T cells, B cells, monocytes, macrophages, eosinophils, neutrophils, dentritic cells, natural killer cells, endothelial cells, and thymocytes) and tissues (liver, intestine, spleen, esophagus, stomach gastric, colon sigmoid, colon mucosa, heart, and pancreas) for which we identified PMDs with MethylSeekR (see Additional file 1 for the complete list of samples). Figure 1 shows methylomes of different cell types with the corresponding segmentation tracks. The lengths of PMDs vary broadly, ranging from 100 kb up to 20 Mb (Additional file 2: Figure S1). PMDs cover a large portion of the genome (50–75%). The average and individual levels of PMD methylation vary between different cell types (boxplots in Fig. 2a). While PMD positions in the genomes are highly conserved across cell types, in general, only roughly 26% of the genome is annotated as completely shared PMDs across all cell types (Fig. 2b). Overall, PMDs are enriched for the broad heterochromatic marks H3K27me3 and H3K9me3 and depleted for the broad euchromatic mark H3K36me3. The latter is also reflected in the low appearance of annotated transcriptional units within PMDs and an overall low average transcription of genes located in PMDs (Fig. 1 and Additional file 2: Figure S2).

To gain a deeper insight into the cell-specific and genome-wide distribution of PMD methylation profiles, we generated and applied a modified ChromHMM [15] approach, “ChromH3M,” as an abbreviation for ChromH**MMm**eta segmentation (see details in the “Methods” section and Additional file 2: Figure S3). In brief, we bin the genome into 1 kb tiled windows, labeled as 1 or 0 according to the presence/absence of PMDs for each sample. This binarized signal is then processed with ChromHMM to generate a 15-state model. The emission probabilities are displayed after hierarchical clustering. This approach generates PMD clusters discriminating cell type origin and/or cell-related subgroups (Fig. 2a). Only five out of 171 samples did not cluster together with samples of similar origin. This approach is surprisingly stable even across cells which differ strongly in their overall methylation level (shown as box plots in Fig. 2a). We also used shorter LMR and UMR regions for such a ChromH3M meta-segmentation and roughly obtained the main subgroups in hierarchical clusters using 10,000 bootstraps and an “au” threshold of 97 (see Additional file 2: Figure S4 and the “Methods” section for details). We conclude that PMDs are strong cell-type-specific discriminators comparable with regulatory changes in short UMRs/LMRs.

For 171 available human methylomes of tissues and primary cells, ChromH3M generates a tree with six main branches separating myeloid from lymphoid cells, endothelial tissues, liver tissue, and tissues of the digestive system and heart. Myeloid cells split into two subclusters: the granulocytes (neutrophils and eosinophils) and agranulocytes (monocytes, macrophages, and dentritic cells). Members of both subclusters have similar average PMD methylation. B cells and T cells form a lymphocyte cluster which branches off into subgroups of memory T and B cells, i.e., central and effector memory T cells, germinal center and memory B cells, respectively. This indicates that cell types not only display a distinct overall PMD topology but also acquire distinct PMD substructures upon proliferation and differentiation [13].

We furthermore observe that, in general, PMDs have extended heterochromatic signatures in both primary cells and permanent cell lines (Fig. 2c). PMDs cover relatively gene-poor regions with mostly lowly/unexpressed genes (Additional file 2: Figure S2). The ChromH3M analysis reveals a couple of distinct features of cell-type-specific PMDs (Fig. 2a). For instance, states 10 and 11 comprise regions that only are HMDs in liver and endothelial cell types, respectively. State 4 discriminates myeloid HMDs from PMDs in other cell types. State 3 defines B and T cell-specific PMDs (Fig. 2a, b). The shared PMDs are defined by states 1, 8, and 9, while states 14 and 15 define shared HMDs.

To explore the biological functions of genes present within cell-type-specific HMDs/PMDs, we performed a functional annotation analysis with DAVID [16, 17] for genes in state 10 and state 3. For the former, liver-specific HMDs, the GO terms liver tissue expression, Rotor syndrom disease (lack of hepatocyte pigment deposits), and the KEGG pathway for drug metabolism through cytochrome P450 were obtained. These genes exhibit significantly higher expression in liver tissue/hepatocytes than in other cell types (two-way ANOVA and Tukey HSD post hoc test, *p* adj = 0) (Fig. 2d). Furthermore, these HMDs are largely devoid of heterochromatic marks and enriched for the transcriptional elongation mark H3K36me3 across gene bodies (Additional file 2: Figure S5, left panel). This is exemplified by two hepatocyte-specific gene loci CYP2B6 and FMO6P (Additional file 2: Figure S6). The latter state, number 3, marks B and T cell-specific PMDs. Hence, these regions in B and T cells are enriched with the repressive mark H3K27me3 and, to a lower degree, with H3K36me3. Further, the functional analysis provides cell-type-associated terms, cell differentiation, inflammatory response, adaptive immune response and specific surface antigen MHC class I, in addition to the KEGG pathway for the hematopoietic cell lineage. Interestingly, the expression levels of these genes are downregulated in accordance with their PMD annotation. However, regarding only the DNA methylation signal, there is a trend to split the B and T cells into naive versus memory cells. This discrimination can neither be confirmed by ChIP-seq nor by RNA-seq (see Additional file 2: Figure S5, right panel). This could be due to the limitation in detecting the precise boundaries of shallow PMDs in naive cells.

In summary, the ChromH3M results indicate a domain-wide transition of cell-type-specific PMDs into HMDs and vice versa along with transcriptional regulation. The direction of this transition couples with specific changes in heterochromatic states.

A ChromH3M analysis on 24 WGBS mouse samples (Additional file 2: Figure S7) shows a similar classification and distribution of PMD states, confirming that our findings not only hold for human but describe a feature apparently conserved among mammals. In mouse, we identify cell-type/tissue-specific PMDs for neuron, intestine, colon, and mammary epithelial cells. Furthermore, the epithelial cells group into cells of the luminal and the basal compartment. We conclude that in human and mouse, PMDs are excellent epigenome classifiers of cell-type-specific topologies.

### Chromatin compaction increases with DNA methylation erosion at PMDs in immortalized cells

**Fig. 3:**
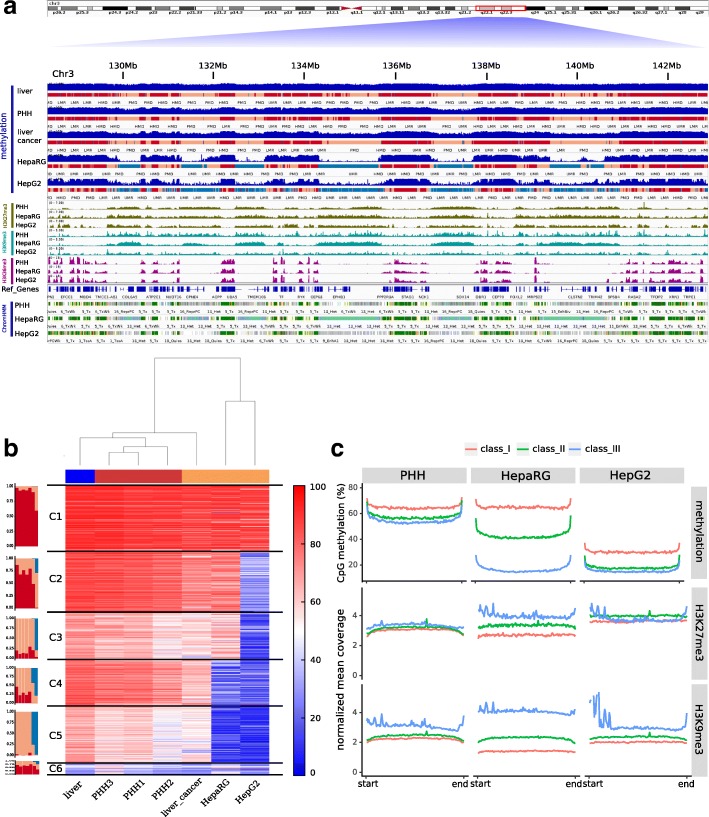
Heterochromatization accompanied by DNA methylation erosion at PMDs in cancers. **a** A snapshot of 14 Mb of chr3 showing the relevant epigenetic marks. Top: distinct DNA methylation tracks and the MethylSeekR segmentation of liver tissue, isolated hepatocytes (PHH), liver cancer tissue, HepaRG, and HepG2 cell lines, respectively. PMDs of primary cells and normal and cancerous tissues are extensively and selectively less methylated in cancer cell lines (largely converted into unmethylated regions). Middle: histone marks H3K27me3, H3K9me3, and H3K36me3 in the same samples. Bottom: ChromHMM segmentation based on these three histone modifications in addition to H3K4me3, H3K4me1, H3K27ac, and Input (see details in the “Methods” section). **b***K*-means clustering (*k* = 6) based on the averaged methylation in 10 Kb bins. Cluster 1 represents the most (almost fully) methylated bins across all samples, while the other clusters are ordered according to the progressive erosion of methylation in PMDs. Bar plots (left) beside the heatmap show the percentage of the annotated bins as HMD, PMDs, and UMR for each sample in each cluster. **c** Progressive change of DNA methylation in PMDs across cancer cell lines. The top of the figure shows classified and grouped PMDs (three classes) based on the average PMD methylation levels in PHH and their corresponding overall levels in HepaRG and HepG2, respectively. Note the intermediate status of HepaRG, e.g., with a higher similarity to PHH in class_I (most highly methylated), an intermediate status in class_II and a higher similarity to HepG2 in class_III (lowest methylation level). The bottom shows the PMD wide changes in heterochromatic marks across the clusters defined by DNA methylation. The inverse correlation to DNA methylation is most obvious for HepaRG (class_I and class_III)

Immortalized cell lines are widely used for studying cellular mechanisms including the influence of epigenetic control. However, it is known that cells in culture undergo drastic epigenetic alterations linked to passaging and cell replication numbers [18]. To investigate the epigenome-wide changes occurring between primary cells and immortal cell lines, we compared the methylomes of primary cells and cell lines of the same origin. With this comparison, we wanted to monitor the impact of cultivation and cancer-specific changes on PMD formation. We generated epigenome data for isolated primary hepatocytes (PHH) and two hepatic cancer cell lines: the hepatic progenitor cell line (HepaRG) and the liver hepatocellular carcinoma cell line (HepG2). We also include in our comparison results on publicly available liver cancer cells and noncancerous liver tissues (Fig. 3a).

First, we calculated the average methylation across the samples in 10 Kb bins. We then performed *k*-means clustering forming six disjoint clusters which were subsequently annotated by the MethylSeekR segmentation (Fig. 3b). Cluster 1 defines highly methylated bins across all samples while the other clusters show progressive loss of methylation in the order: liver tissue >PHH >liver cancer >HepaRG >HepG2. Interestingly, primary liver cancer was more similar to the non-cancerous liver and PHH than to the cancer cell lines HepaRG and HepG2. Both cancer cell lines have lower methylation levels compared to the primary cells, as seen in clusters 4 and 5. This indicates a different epigenomic pattern in cultivated cancer cells in comparison to primary cancer cells. To gain deeper understanding of the features governing the development and changes in cell-type-specific PMDs, we focused on the analysis of liver PMDs that exhibit changes in the average methylation level in the cancer cell lines. We first extracted PMDs of PHH cells, which exhibit large overlap with PMDs of liver tissue and liver cancer tissue (data not shown). Such primary liver cell PMDs split into three subclasses, with respect to changes in DNA methylation in HepaRG and HepG2 (Fig. 3c). In the first subclass (class_I, red), PHH and HepaRG exhibit the same average degree of methylation (65%), but show a very low methylation state in HepG2. In the second class (class_II, green), HepaRG methylation levels are intermediate between PHH and HepG2, while in the third class (class_III, blue) both HepaRG and HepG2 show the same low average methylation as compared to the primary cells.

Along with the progressive loss of DNA methylation in these three subclasses, we observe a distinct gain of heterochromatic marks (Fig. 3c), suggesting a compensatory effect. The effect is most obvious in the HepaRG cell line which shows an intermediate level of PMD methylation. Moreover, H3K36me3 is positively correlated with DNA methylation across the gene body in the three subclasses (Additional file 2: Figure S8). We confirmed this observation by calculating the average methylation across ChromHMM segments of HepaRG (Additional file 2: Figures S9 and S10). PMDs associated with stronger transcription are higher methylated, on average, and marked by lower levels of heterochromatic marks.

We conclude that in immortalized cells, a progressive erosion of DNA methylation mainly in PMDs is linked to a substantial gain of heterochromatic marks. This is likely to be accompanied by differences in chromatin compaction and regulation in the immortalized cells with a prolonged proliferation. The conversion of PMDs and sometimes of HMDs, found in cancer tissues, into low methylated domains as seen for HepG2 indicate that epigenetic changes found in model cell lines should be interpreted with great care, as they may reflect the properties more of the cell’s proliferation history and less of the cancer state or cell-specific origin.

### Distinct heterochromatic signatures of PMDs predict replication timing

**Fig. 4:**
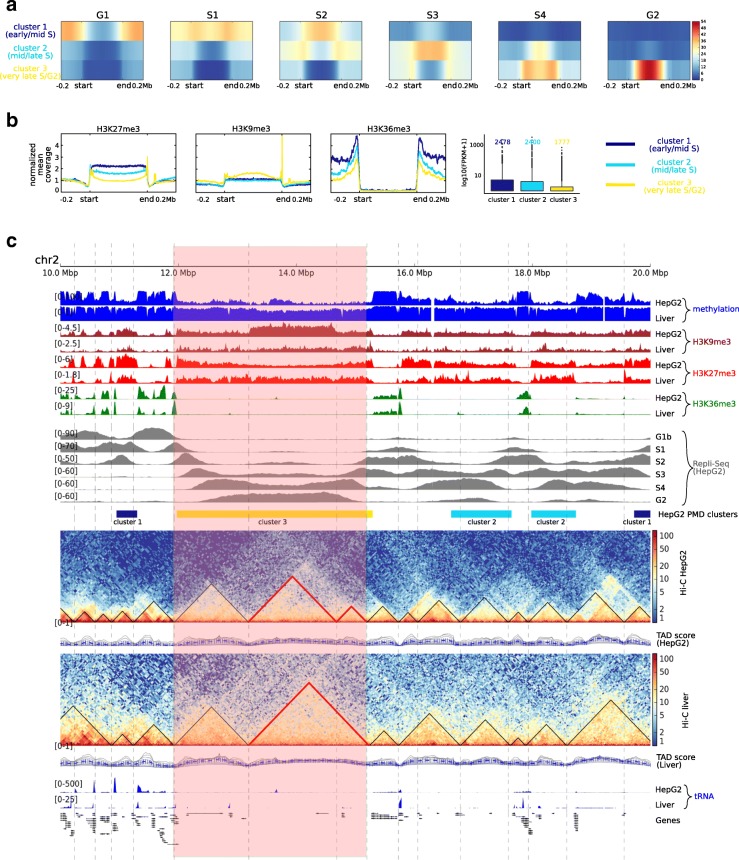
Distinct heterochromatin signatures of PMDs predict replication timing. **a** HepG2 PMDs are classified into three classes according to replication timing signals: cluster 1 represents the early/mid S phase associated with PMD boundaries, cluster 2 represents the middle/late S phase (S3/S4), and cluster 3 represents the very late S phase (S4) and G2. **b** Epigenetic mark signatures across clusters in **a**; H3K27me3 is highly enriched in cluster 1 and in the PMD boundaries of cluster 3 and less so in cluster 2. H3K9me3 enrichment is similar in cluster 1 and cluster 2 and become more prominent in cluster 3. The elongation mark H3K36me3 is depleted in all clusters. PMDs in cluster 3 have the lowest methylation level among the other two clusters and encompass the transcriptionally inactive genes. **c** Different epigenomic data tracks from chr2 shown in the following order: DNA methylation profiles, H3K9me3, H3K27me3, and H3K36me3 histone marks of HepG2 and PHH, replication timing signals (G1-G2) of HepG2, clustered HepG2-hypomethylated/PMDs according to **a**, Hi-C contact matrices of HepG2, and liver with the corresponding called TADs, tRNA, and RefSeq genes. The highlighted region shows one long PMD, roughly 3Mb, extends over three TADs which are splitting according to H3K9me3 signal enrichment. Two of these TADs, marked in red, fuse into one TAD in the liver sample in agreement with the H3K9me3 signature

It has been shown that during cell division, late-replicating regions can become gradually demethylated [19] and long PMDs show widespread H3K9me3 marks bordered by H3K27me3, whereas shorter PMDs are enriched by H3K27me3 only [6]. So far, these features have not been deeply investigated and analyzed in an integrated fashion, i.e., combining DNA methylation and chromatin marks. Using replication timing data for HepG2 from the ENCODE project [20, 21], we clustered HepG2 hypomethylated/PMDs regions (longer than 300 kb), by the *k*-means algorithm, into three clusters (see the “Methods” section and Fig. 4a). We observe that these clusters display distinct combinations of histone modification and DNA methylation (Fig. 4b and Additional file 2: Figure S11). Cluster of early/mid S phase (dark blue) is associated with shortest PMDs, and the chromatin is enriched for the two repressive marks H3K27me3 and H3K9me3. Mid/late S phase cluster (light blue) comprises longer PMDs which are less highly enriched for H3K27me3 compared to the previous cluster. In very late S/G2 phase cluster (yellow), PMDs extend over very long regions making up roughly 50% of the total PMDs/hypomethylated regions (Additional file 2: Figures S12 and S13). These PMDs are strongly enriched for H3K9me3, bordered by H3K27me3. We confirm our clustering by using the three broad histone modification signals in PMDs as predictors and observe a high average prediction accuracy of 0.77 for HepG2 (0.81 for IMR90) (notice that this is a three-class prediction, details in the “Methods” section). The very late replicating regions S/G2 phase have the highest prediction accuracy, suggesting a distinct chromatin signature in this phase. These findings extend previous results [19] indicating that combinations of heterochromatic marks and DNA methylation define early and middle S-phase and late S-G2 phase (Fig. 4b).

### PMDs organization and topologically associated domains (TADs)

To analyze the overall relationship between PMDs and TADs (topologically associated domains), we generated Hi-C data for HepG2 and used available liver Hi-C data [22]. Using HiCExplorer tool [23], we identified 3217 and 4021 TADs in liver and HepG2, respectively. As a consequence, TADs of HepG2 are shorter than those in liver (Additional file 2: Figure S14). This finding is in agreement with Taberlay et al. [24], who showed that cancer cells in general form smaller TADs and establish new boundaries. We find that TAD borders are significantly closer to PMD borders as compared to randomized test borders (*p* value <2.2e −16, Wilcoxon test) (Additional file 2: Figure S15). Moreover, 94% of base pairs within PMDs, in HepG2 and liver, are also annotated as heterochromatic TADs (Additional file 2: Figure S16) (details in “Methods” section). The light red box in Fig. 4c highlights a typical example of a region in which several TADs exist in HepG2 and primary liver that are belonging to one PMD. In HepG2, we observe the formation of an extra TAD (marked in red) but not the formation of an additional PMD boundary. This extra TAD shows a strong enrichment of H3K9me3.

## Discussion

Our comprehensive integrated analysis of primary human cells adds valuable novel insights into the structure and function of PMDs in human and mouse epigenomes. Building on our first report of PMD changes in primary T cells [13], we here integrated publicly available WGBS datasets fulfilling high-quality standards to systematically analyze the features of PMDs in primary cells, primary tissues, and immortalized cells from human and mouse. We apply a new integrative “ChromH3M” approach which combines existing tools and represents an easy and straightforward method for analyzing and integrating a large cohort of WGBS datasets. This allowed us to define and compare PMDs across hundreds of WGBS samples, revealing a couple of intriguing new DNA methylation properties with respect to genome organization, timing of DNA replication and cell-type-specific gene regulation.

We find that PMDs comprise up to 75% of all epigenomes. However, only roughly 26% of the genome consists of PMDs that are shared across all investigated cells (shared PMDs). These common/shared PMDs have also been a focus of a recent analysis by Zhou et al. [25] confirming some of our findings. As a major difference, we also find that PMDs serve as excellent cell type classifiers as cells with functional similarities show a more similar PMD arrangement and topology, arguing for a shared developmental origin of lineage-specific PMDs. Finally, we observe that PMD methylation changes in some cells when they proliferate and show strong methylation decreases in immortal cell lines.

Analyzing the PMD topology in more detail, we observe that the epigenomes of cells are partitioned into long regions of PMDs interspersed with HMDs. These two classes of epi-domains show contrasting chromatin signatures. While PMDs are more heterochromatic and gene-poor regions, HMDs show strong transcriptional activity and enrichment of genes. This finding generalizes previous isolated observations reported by [4, 8, 10, 13, 26] to a number of different cancer types and cell lines. We also find cell-type-specific changes from PMD to HMD, and vice versa, occurring in genomic regions that contain genes functionally enriched for cell-type-specific properties. This finding points towards a developmental control of PMD and HMD formation. The complete understanding which partitioning of HMDs and PMDs defines a precursor ground state of a cell type needs more investigation. Such knowledge will help understanding the role of epigenetic domains in cell differentiation.

We find that long PMDs have a lower density of protein-coding genes, lincRNA, and pseudogenes relative to the shorter PMDs. In general, protein-coding genes are less highly expressed in long PMDs than in shorter PMDs and HMDs (Additional file 2: Figures S17 and S18). We hypothesize that this is also reflected in the more pronounced constitutive heterochromatic nature of long PMDs as compared to the more facultative heterochromatic nature of shorter PMDs [27]. Shorter PMDs retain more epigenomic plasticity with more pronounced cell-type-specific features.

PMDs can also be divided into different subclasses which are observed in different stages of DNA replication. A hallmark of the late stages of replication (S4 and G2) is their length and the presence of the constitutive heterochromatic mark H3K9me3 together with a characteristic enrichment of H3K27me3 at the boundaries. On the other hand, the early/middle S phases (S1-3) PMDs are shorter and exhibit a higher overall proportion of H3K27me3. The length-dependent histone modification pattern is in agreement with previous findings in medulloblastoma [6]. The differences are strong enough to be used as predictors of the replication phases. Our results are consistent with a previous report [19] and extend its results by providing a detailed characterization of chromatin state and DNA methylation at PMDs in relation to cell cycle. Moreover, the long PMDs associated with the late S4-G2 phases overlap with 56% of the bases within shared PMDs. The shorter PMDs retain a greater variability, confirming our hypothesis that shorter PMDs possess epigenetically more less rigid heterochromatic structures than longer ones. This characteristic could be relevant for differentiation, cell-fate determination, and/or cell maturation processes.

To deeper understand the cell-type-specific changes occuring at PMDs (and HMDs), in cancer and cancer cell lines, we compared the DNA methylation landscape of primary human hepatocytes (PHH) to liver cancer tissue and hepatocellular carcinoma cell lines (HepaRG and HepG2). Notably, the methylome of primary liver cancer retained a PMD structure highly similar to primary cells. PMDs in cancer tissue show a mild but clearly reduced level of methylation. In cancer cell lines, however, the DNA methylation in PHH-specific PMDs strongly decreases. The regions with lower methylation still retain the typical PMD histone marks even if they are completely unmethylated. So far, we have no explanation to how this aligns with models suggesting that global demethylation is caused by a global loss of heterochromatic marks such as H3K9me2/3 and consequently a lack of UHRF1 activity during replication by deregulation of DNMT1 [28]. When counting the unmethylated regions as PMDs, the overall PMD structure of cell lines is hepatocyte-like. It is likely that the strong erosion of DNA methylation is the consequence of extensive cultivation leading to a proliferation-dependent loss of methylation while maintaining or even enforcing heterochromatic marks such as H3K9me2/3. An alternative hypothesis is that the loss of PMD methylation is caused by the selection/expansion of cell subpopulations with lower methylation. To better understand the generation of increased demethylation in PMDs as a consequence of cell proliferation (cell division), we performed an experiment outlined in Additional file 2: Figure S19A. Human T memory cells were obtained from three different donors, and from each, such “bulk” sample single cells were isolated and clonally expanded following TCR stimulation. After proliferative expansion, single clonal cultures were analyzed and compared to the starting “bulk” samples (mixed T cells) by RRBS. We observe a preferential loss of methylation in PMDs (Additional file 2: Figure S19B, confirming our results reported in Durek et al. [13]). We calculated the percentage of fully methylated, fully unmethylated, and mixed patterns of four consecutive CpGs within single read (see Additional file 2: Supplementary methods for details). Interestingly, the fraction of mixed patterns within PMDs remains constant in all three single cell clones while the fraction of fully unmethylated patterns expands and the fraction of fully methylated patterns diminishes (Additional file 2: Figure S19C). Moreover, 20–35% of CpGs in the fully methylated fraction loses methylation in all three clonally expanded populations. This strongly argues for a gradual loss of methylation coupled to cell division rather than a clonal selection considering that the analyzed cell populations arise from three independent single cells/donor. Overall, our findings are in agreement with result of a recent paper [29] which suggested that at least in cancers, hypomethylation is unlikely to be the result of a “population level effect” only and the extent of hypomethylation is proportional to the cell division rate of the tissue.

A genome-wide decrease of methylation is also seen in early human and mouse embryos [30–34]. Schroeder et al. [35] reported that PMDs can be detected in the oocyte and early embryos of several species but that they are not detectable in placentae, a tissue that shows a low level of overall DNA methylation. Upon differentiation, the genome-wide DNA methylation levels (also in PMDs) increase in somatic cells probably to prevent genomic and transcriptional instability that is observed in fast proliferating cancer cells that usually show a pronounced erosion of PMDs [11]. These findings are in line with our analysis suggesting that while PMDs are general features of (adult) somatic cells, proliferation, differentiation, and development have an impact on PMD topology and genome-wide epigenetic memory.

In general, levels of PMD methylation should be considered when comparing local epigenetic states in vitro particularly when comparing healthy and cancerous tissues to immortal cell lines. In our recent study [13], we suggested a way to consider such global demethylation effects for the detection of differentially methylated region (DMR). Here, we screened for DMRs based on their deviation from the global methylation change rather than applying a fixed cutoff (for more details, see [13]). DMRs were stratified over PMDs and HMDs such that many DMRs simply following the global change of methylation could be excluded. In B cells, this procedure reduced the number of DMRs within PMDs tremendously (from 28,014 to 8338 using adaptive filtering when comparing naive B cells with plasma cells). On the other hand, DMRs, within PMDs, that gain DNA methylation upon differentiation are increased (2811 DMRs in comparison to 95 retrieved by basic thresholding method) (Additional file 2: Figure S20). Genomic region enrichment analysis for such DMRs using GREAT [36] provides cell differentiation and development relevant as major terms (Additional file 2: Figure S20). These findings demonstrate the advantage of stratifying DMRs according to increasing and decreasing of DNA methylation in HMDs and PMDs, affording more insight into the biological role of the genes associated with these DMRs.

A very important observation is that PMD and HMD prediction can be used as a proxy for and/or support Hi-C data when detecting and classifying TADs. When overlaying TAD and PMD predictions, we observe that they largely co-localize and often share the same boundaries. Specifically, PMDs almost completely overlap with heterochromatic TADs. However, we also observe that multiple TADs can overlap with one single PMD. This suggests that either PMDs cover domains larger than TADs or indicates that Hi-C data provide a more fine-grained resolution for domain boundaries. Overall, we observe that there are commonalities as well as differences when aligning TADs and PMDs, and their topological organization and functional relation will have to be further investigated to better understand their dependencies. A recent study by Nothjunge et al. [37] showed that the establishment of heterochromatic (B) compartments precedes PMD formation. As this study only focused on DNA methylation, it remains an open question if B compartments are indeed established prior to a heterochromatic domain formation which we see as one feature of cell-type-specific PMDs.

## Conclusions

We provide a comprehensive analysis of PMDs for 195 human and mouse methylomes including more than 157 primary cell samples. Our analysis adds a new dimension to studying DNA methylation on a large scale extending beyond the context of cis-regulatory elements that has been studied extensively. Our results show that PMDs are an excellent classifier of cellular origin and confirm that they are indicators of the cellular proliferation history. In addition, PMD heterochromatic histone mark signatures serve as an effective classifier for distinguishing early from middle and late replication domains. ChromH3M is an easy and straightforward framework for integrated analysis of large-scale WGBS data and can highlight specific combinatorial patterns of PMDs across large number of samples. PMDs are also a useful adjusting tool for detecting functional DMRs in highly proliferative cells. We believe that PMDs are a crucial epitopological signature beside their role in gene regulation. Our analysis reveals an important limitation in using cultivated cells for disease-associated epigenetic studies as they undergo strong changes in their epigenetic topology.

## Methods

### WGBS

Coverage and methylation fraction of human samples were downloaded from the Roadmap Epigenomic Project http://egg2.wustl.edu/roadmap/data/byDataType/dnamethylation/WGBS. Blueprint data was downloaded from ftp://ftp.ebi.ac.uk/pub/databases/blueprint/data/homo_sapiens/GRCh38/ and then mapped to hg19 using liftOver from UCSC [38]. DEEP data was taken from previous studies [13, 39]. Bed files containing the coverage and methylation levels at CpG resolution from [40] were directly used in the analysis. We list all samples with the relevant sources in the Additional file 1.

### MethylSeekR segmentation

All samples were segmented into partially methylated domain (PMDs), lowly methylated regions (LMRs), and unmethylated regions (UMRs) using the MethylSeekR tool [3]. The rest of the genome, excluding gaps as annotated by UCSC [38], was denoted as highly methylated domains (HMDs). We ran MethylSeekR with default parameters: a coverage cutoff at five reads per CpG, methylation level threshold at 0.5, and maximum FDR of 0.05 for detection of hypomethylated regions, resulting in a threshold of at least four CpGs per LMR, 101 CpGs per sliding window nCGbin = 101, and smoothing over 3 CpGs. Methylation levels of both strands were aggregated and weighted average methylation levels were plotted as box plots across PMDs.

### ChromH3M segmentation

In order to explore PMDs and find combinatorial patterns across samples, we binned the genome into 1 kb windows and annotated each of them with 1 if the bin overlaps with a PMD and 0 otherwise across all samples. We used ChromHMM [15] to train this binarized signal with a 15-state HMM. We termed this method “ChromH3M.” The emission probabilities and states were hierarchically clustered using Euclidean distance and ward.D2 as an agglomeration method in the R environment [41]. The very same analysis was performed for LMRs and UMRs, respectively. To assess the uncertainty in the hierarchical clustering, we calculated an unbiased *p* value (AU *p* value) via multiscale bootstrap resampling (*n* = 10,000). The two cell line samples HepaRG and HepG2 were not included in ChromH3M analysis.

The normalized mean coverage of three broad histone marks (H3K27me3, H3K36me3, and H3K9me3), generated by the DEEP pipeline http://doi.org/10.17617/1.2W [42], were plotted genome-wide across the PMDs with proper flanking regions using deepTools [43]. The number of protein-coding genes falling within PMDs was calculated, demanding a minimum of 80% of the gene length to be overlapping with the segment. A pseudocount of 1 was added to FPKM to avoid zeros in the box plots.

The heatmap in Fig. 3b was generated by binning the genome into 1 kb windows and averaging the methylation levels across all samples resulting in roughly 280,000 windows which then were clustered by k-means into six clusters and annotated with methylSeekR segments. Samples were hierarchically clustered with ward.D2 and Euclidean distance. Sex chromosomes were excluded from the aforementioned analyses.

### Clustering of PHH PMDs and cancer cell lines

PHH PMDs shorter than 20 kb were filtered, and a matrix of methylation levels in 1 kb windows across PHH, HepaRG, and HepG2 was calculated after normalizing all PMDs to the same length of 150 kb using deepTools [43]. The windows were clustered with *k*-means method into three clusters. H3K27me3, H3K9me3, and DNA methylation signals were plotted along PMDs of each cluster using deepTools [43].

### Analysis of replication domains

Replication timing signals were downloaded from ENCODE project and used directly (details about this data are available from). A two-state HMM was used to segment the HepG2 methylation profile into highly methylated and PMDs/hypomethylated regions using the “HiddenMarkov” R package [44], assuming that each CpG may have one of the two states: foreground state with high methylation level and background state with low methylation level. Regions shorter than 300 kb were filtered. The mean coverage of replication signals (G1, S1-S4, and G2) was calculated in 1 kb bins across normalized (to 500 kb length) and flanked PMDs (250 kb up and down-stream) using deepTools [43]. PMDs were then clustered using *k*-means into three classes: early/middle S phase, middle/late S phase, and late S/G2 phase. The mean coverage signals of H3K27me3, H3K9me3, and H3K36me3, and DNA methylation levels were plotted across the PMDs of each class using deepTools. The number of protein-coding genes falling into each class was calculated, demanding 80% of the gene length to be within the PMD. FPKM values were plotted as box plots in log scale with pseudocount of 1 to avoid zeros. For the prediction of replication domains, we built a multiclass classification model using the counts of reads of each histone mark in 1 kb bins as predictors and the three aforementioned clusters as response at each PMD. We split the data into 75% training set and 25% test set. We trained the model with a random forest classifier and selected the model using 10-fold CV repeated five times. The prediction accuracy was calculated based on the confusion matrix between the predicted and the reference values. One-versus-all accuracy was calculated and then the average accuracy was calculated. This analysis was performed using the caret package https://github.com/topepo/caret/ in the R environment. The analysis of genomic regions regarding DMRs, with and without adjusting for the global DNA demethylation, was carried out using the GREAT tool [36]. GO analysis was done using DAVID [16, 17].

### Chromatin state segmentation

All Chip-Seq samples, listed in Additional file 2, were preprocessed starting from raw BAM files as follows: duplicate reads were removed using samtools version 1.3 with the filter “-F 1024.” Regions of known artifacts (“blacklist regions”) taken from the ENCODE project https://www.encodeproject.org/ [20], which we adapted to account for differences between ENCODE’s hg19 and DEEP’s hs37d5 assembly, were filtered out using bedtools version 2.20.1 with the subcommand “pairtobed” and the option “-type neither.” After preprocessing, the filtered BAM files for all six histone marks plus Input were used as input for the chromatin state segmentation using ChromHMM version 1.11 (Java 1.7) with default parameters. We did not train a dedicated ChromHMM model for our dataset, but used the available ROADMAP 18-state model [45] to benefit from its biologically meaningful state labeling, which enabled us to immediately interpret the chromatin state maps in the context of this work.

### HepG2 Hi-C

HepG2 cells have been fixed for 10 min using 1% formaldehyde in D-MEM and quenched for 5 min in 125 mM glycine. After two PBS washes, cells have been collected by scraping them off the plate and snap-frozen in liquid nitrogen. Hi-C experiments have been conducted as previously described [23], with the following modifications. Nuclei from cell pellets containing about four million of cells have been extracted by sonication [46] using the following parameters: 75 W peak power, 2% duty factor, 200 cycles/burst, and 180 s, using Covaris milliTubes and Covaris E220 sonicator. After nuclei permeabilization, chromatin has been digested overnight at 37^∘^C using HindIII high fidelity (80 units per million cells; R3104S, NEB). Biotin incorporation has been carried out at 37^∘^C for 1 h in 300 *μ*l volume using these reaction conditions: 50 mM of each nucleotide (dATP, dTTP, dGTP, biotin-14-dCTP, from Life Technologies, 19518-018) and 8 U of Klenow (NEB, M0210L). Ligase mix has been added to each sample followed by 4 h of incubation at room temperature under rotation. After nuclei lysis, protein digestion and overnight de-crosslink, DNA has been precipitated and sonicated to 100–600 bp. Biotinylated DNA has been pulled down as previously described. One hundred nanograms of DNA bound to beads have been used for library preparation using a modification of the NEBNext Ultra DNA library preparation workflow (NEB, E7370). DNA bound to beads has been end-repaired, A-tailed, adaptor-ligated, and USER-treated following manufacturer’s instruction. After a bead wash, DNA has been eluted from the beads by incubating at 98^∘^C for 10 min. Adaptor-ligated DNA has been PCR amplified using 7 PCR cycles. Libraries have been sequenced paired-end, with a read length of 75 bp, on the Illumina NextSeq 500 instrument.

### Hi-C data processing

Reads were mapped to the human reference genome hg19 (37d5) using bowtie2 [47], and then samtools [48] was used to convert the reads to BAM format. A matrix of read counts over the bins in the genome, considering the sites around the restriction site AAGCTT was built using the hicBuildMatrix function from HiCExplorer [23]. Ten bins were merged with hicMergeMatrixBins and then the matrix was corrected for GC bias and very low/high contact regions. To compute the TADs we first calculated the TAD scores by “hicFindTADs TAD_score” command with the following parameters “–minDepth 300000 –maxDepth 2000000 –step 70000” and then TADs were identified by “hicFindTADs find_TADs” command. The interaction matrix and other signal tracks were also visualized using HiCExplorer.

### Comparison between TADs and PMDs

To test the consistency between TAD borders and PMD borders, we generated an equally sized set of randomized borders and calculated the shortest distance between TAD borders and (i) PMD borders and (ii) the randomized borders. A Wilcoxon test was carried out between the two distance distributions. To calculate the overlap between PMDs and heterochromatic TADs (generated as described above), we classified TADs using histone marks into two classes by *k*-means from deepTools. One class is enriched by heterochromatic marks and the other by euchromatic mark. We counted the number of overlapping base-pairs between PMDs and the heterochromatic TADs and then plotted the results as venn diagrams. The comparison was done for liver and HepG2. In this analysis, PMDs within a distance of 50 kb were fused and only those longer than 300 kb were included. This was done to exclude intersecting LMRs and UMRs.

## Additional files

Additional file 1 List of samples. XLSX sheet with list of samples and the corresponding URLs and accession numbers of the raw data. (XLSX 36 kb)

Additional file 2 Supplementary materials. PDF document with supplementary figures and supplementary methods. (PDF 11,526 kb)

Additional file 3 Review history. (DOCX 54 kb)

## Abbreviations

HMDs: Highly methylated domains
HMM: Hidden Markov model
LMRs: Lowly methylated regions
PMDs: Partially methylated domains
PHH: Primary human hepatocytes
TADs: Topologically associated domains
UMRs: Unmethylated regions
