# Supplementary material for: A comprehensive analysis of 195 DNA methylomes reveals shared and cell-specific features of partially methylated domains
Source: Genome Biol. 2018 Sep 28;19:150. doi: 10.1186/s13059-018-1510-5 (PMC6161375; doi:10.1186/s13059-018-1510-5)
Supplement: Supplementary file 2 — Supplementary materials. PDF document with supplementary figures and supplementary methods. (PDF 11,526 kb) [file 13059_2018_1510_MOESM2_ESM.pdf]

Figure S1

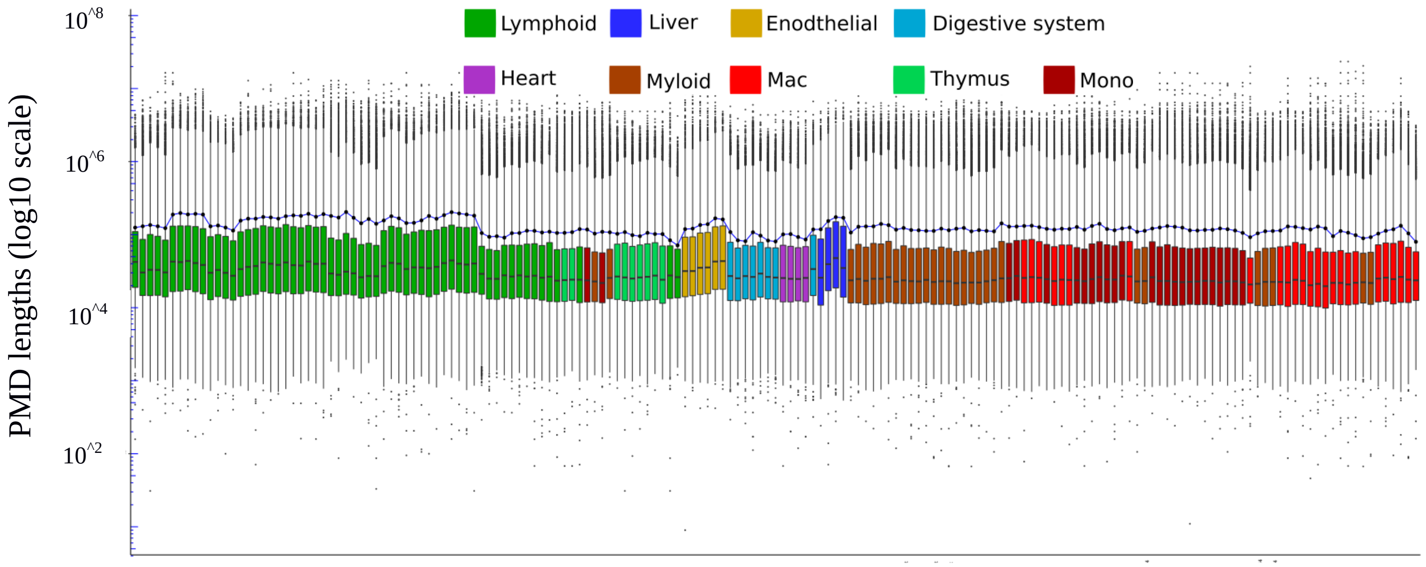

Figure S1. PMDs length distribution. The mean length distribution of PMDs is ~150 kb

Figure S2

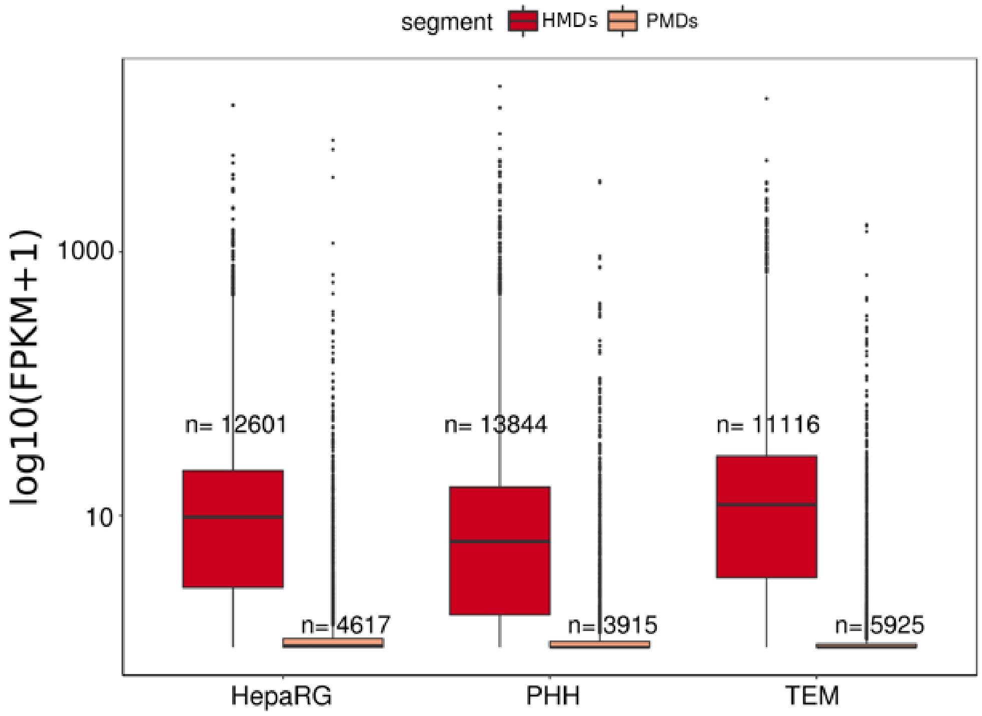

Figure S2. PMDs are gene-poor regions. Number of genes and their FPKM values in PMDs/HMDs in one cell line (HepaRG) and two primary cells (hepatocytes and effector memory T-cells).

Figure S3

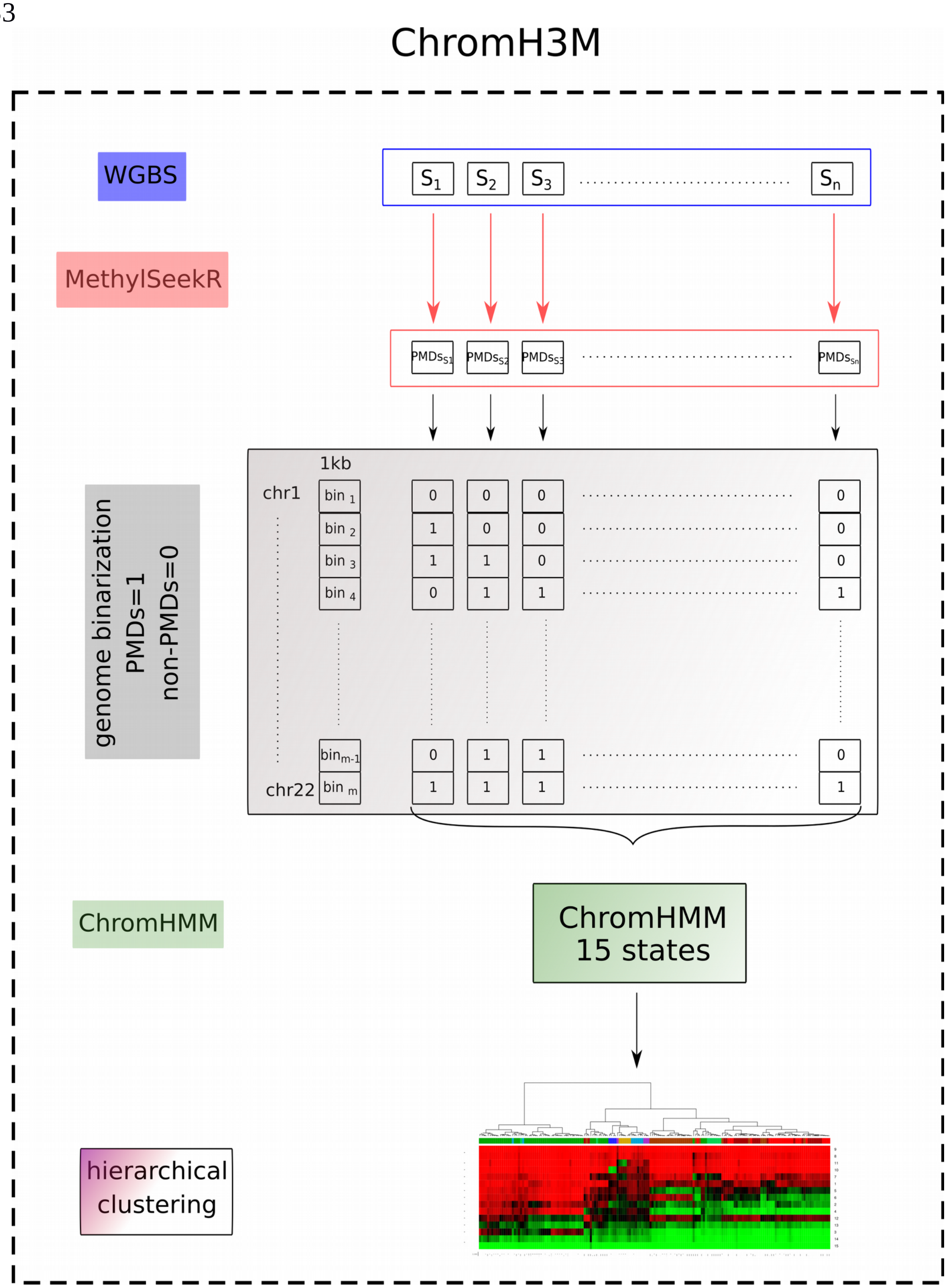

Figure S3. ChromH3M workflow. MethylSeekR is applied to each sample to identify PMDs. 1Kb bins across the genome are annotated with 1/0 according to presence/absence of PMD for each sample. The binarized signal is loaded into ChromHMM and a model with 15 states is trained. The emission probabilities and states are then hierarchically clustered.

Figure S4

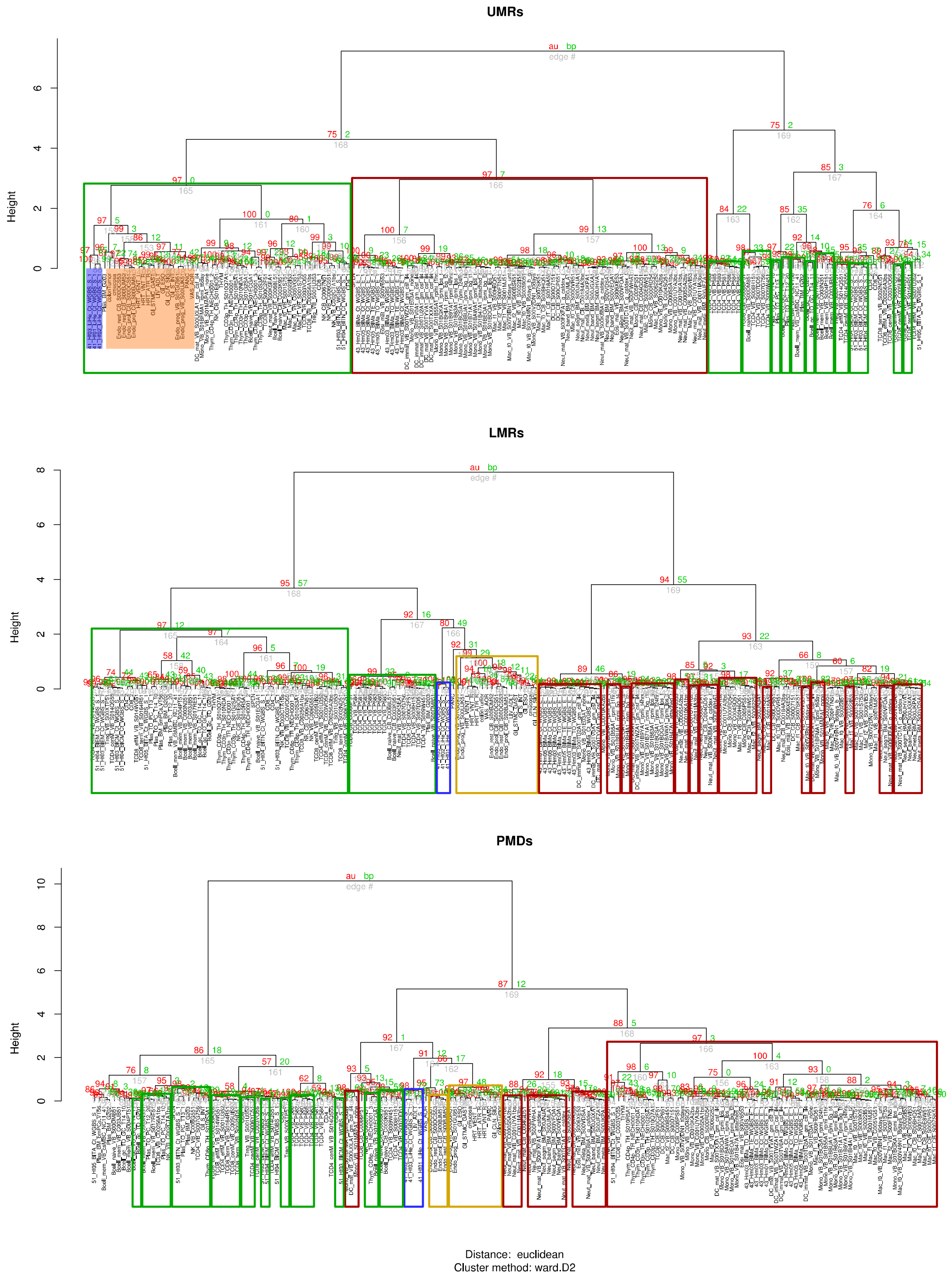

Figure S4. Dendrograms calculated from emission probabilities derived from ChromH3M model for PMDs, LMRs and UMRs analyzed with 10000 bootstrap replications. Red values are AU (Approximately Unbiased) p-value and green values are BP (Bootstrap Probability) value. Colored

boxes are the clusters with AU greater than 97% and they contain the same samples across the analyzed three segments. The two shaded boxes in UMRs correspond to the two same colored boxes (blue and brown) in LMRs and PMDs.

Figure S5

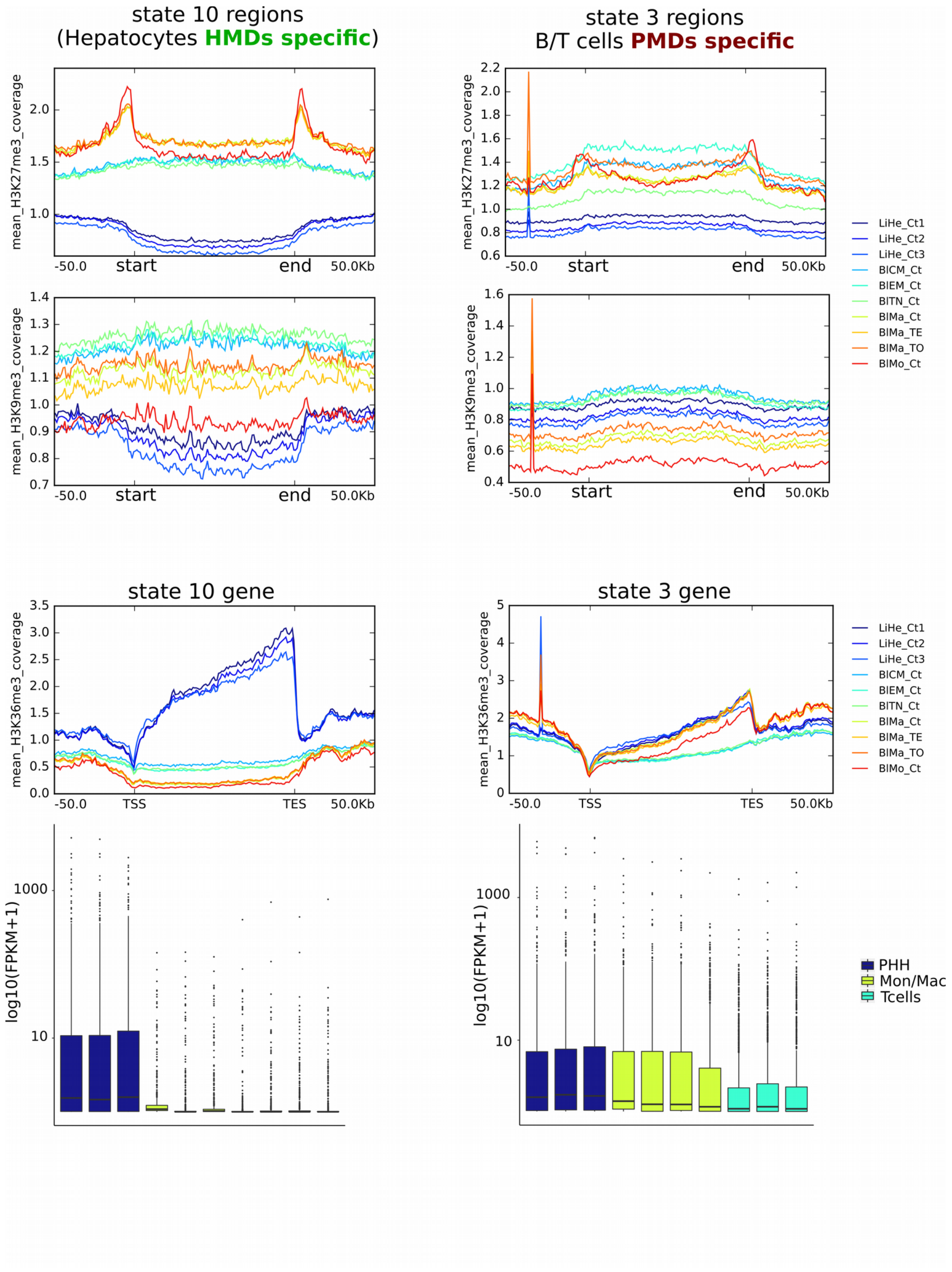

Figure S5. Cell-type specific PMDs/FMRs and their heterochromatic signature. The left panel represents the broad histone mark signals of four cell types; hepatocytes, monocytes, machrophages and T-cells in hepatocyte-specific HMDs together with FPKM values of the associated genes. The right panel is the same but for B/T cell-specific PMDs.

Figure S6

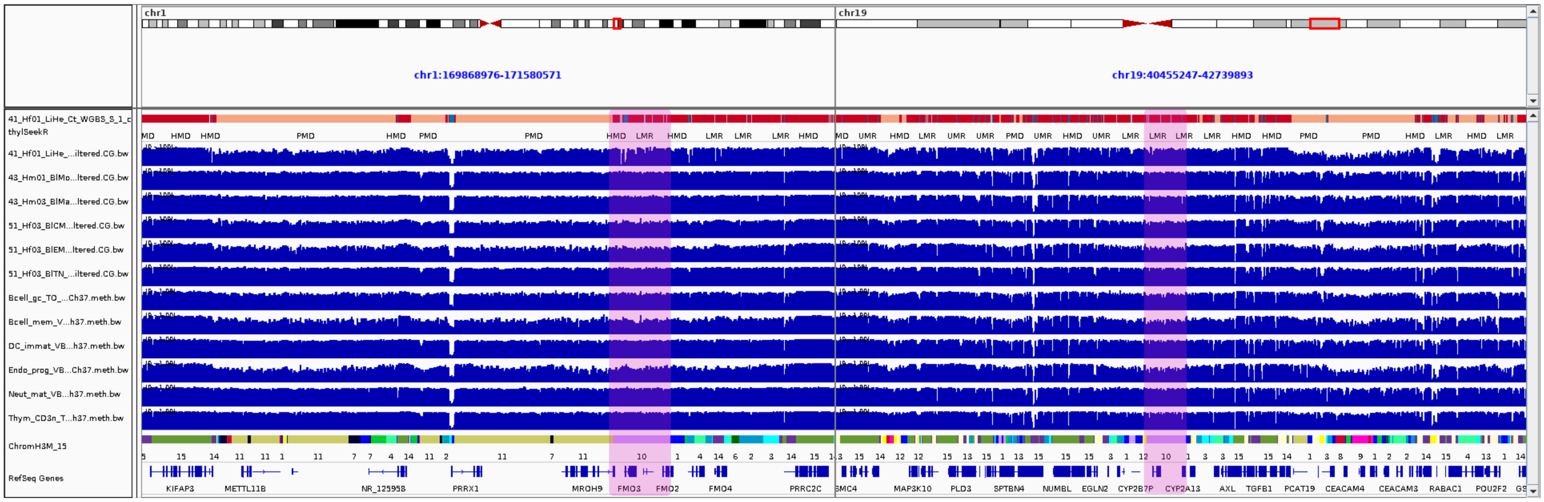

Figure S6. hepatocyte gene specific locus. Two hepatocyte gene families CYP and FMO which have been identified to be part of state10 displayed in Figure 2A.

Figure S7

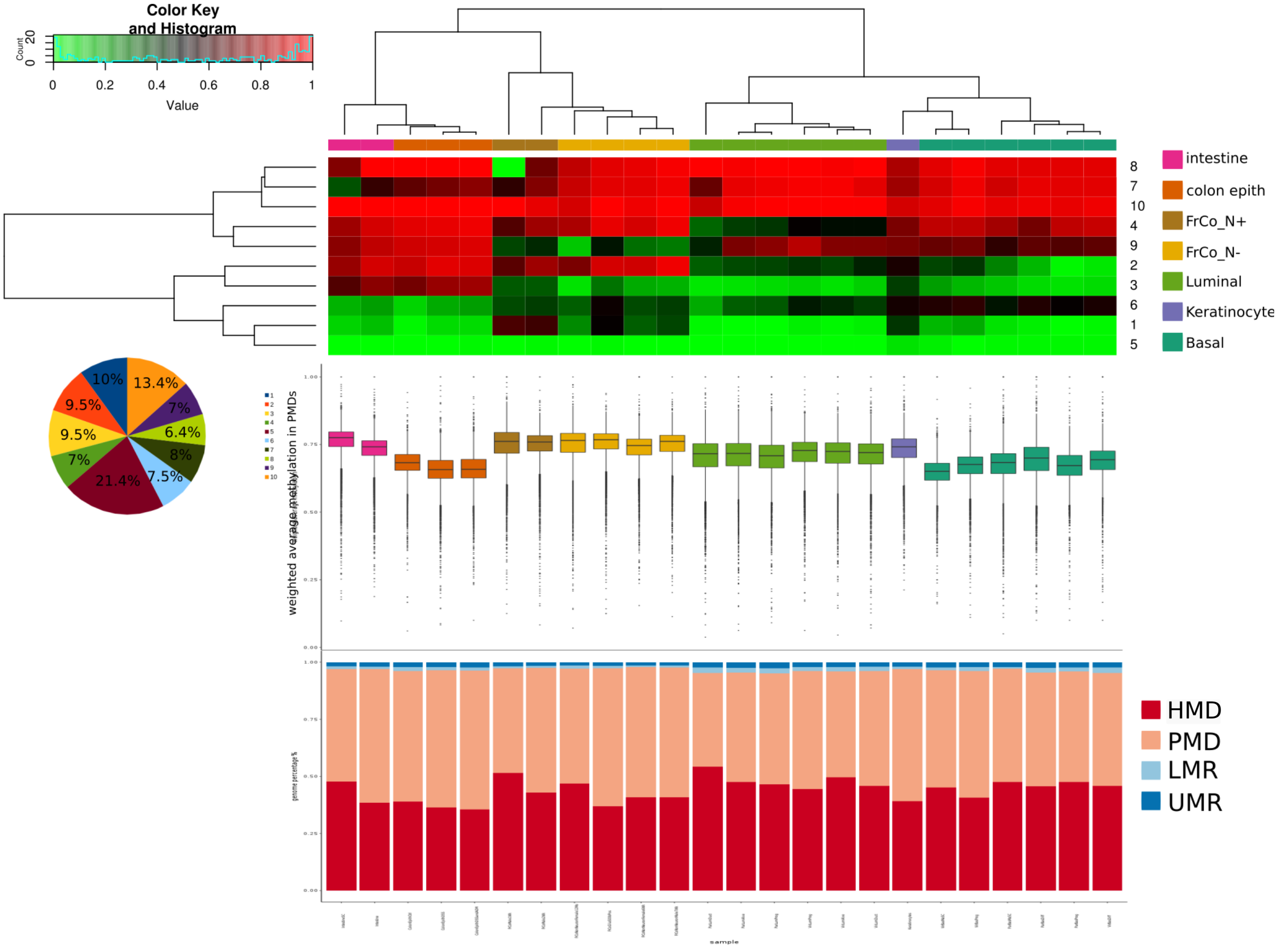

Figure S7. Analogous to Figure 2A for mouse data

Figure S8

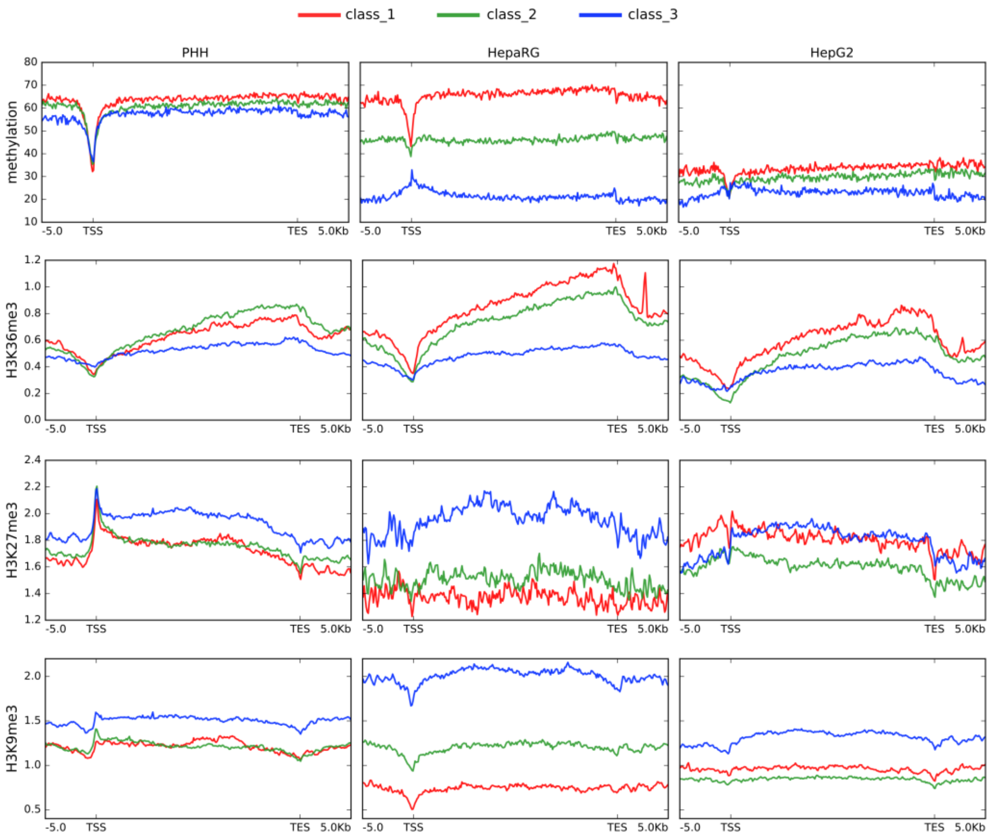

Figure S8. Epigenetic modification signatures in PHH_{PMDs}. DNA-methylation, H3K36me3, H3K27me3 and H3K9me3 signal across the gene bodies in three classes according to Figure 3C.

Figure S9

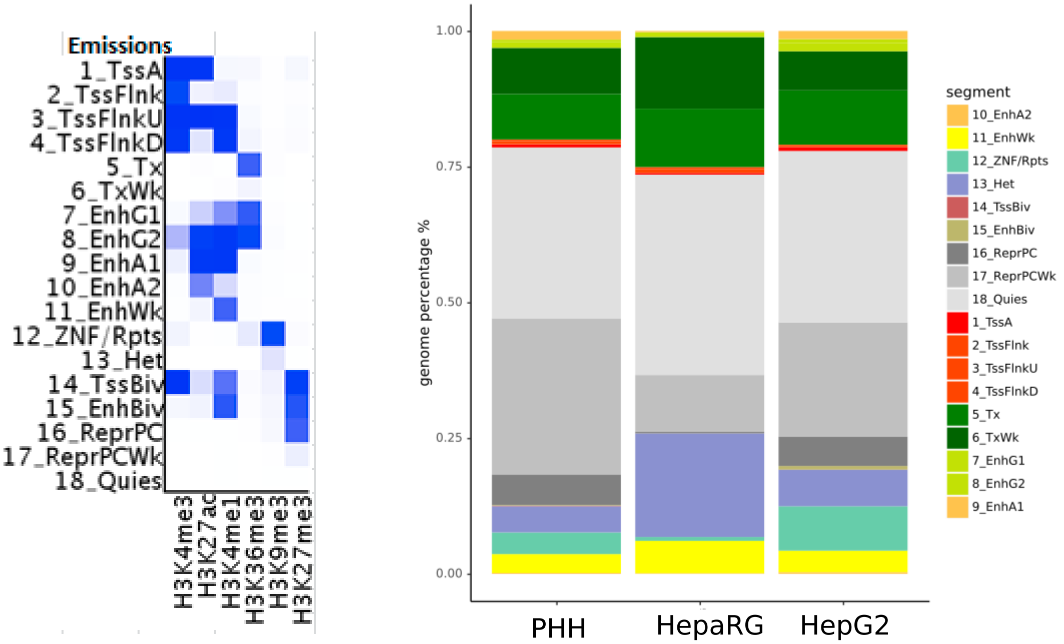

Figure S9. chromatin state segmentation. Emission probabilities of ChromHMM model and ChromHMM segments fraction related to Figure 3A. labels are the following:

1_TssA: Active TSS, 2_TssAFlank: Flanking active TSS, 3_TxFlankU: Flanking TSS Upstream,

3_TxFlankD: Flanking TSS Downstream, 5_Tx: Strong transcription, 6_TxWk: Weak transcription,

7_EnhG1: Genic enhancer1, 7_EnhG2: Genic enhancer2, 9_EnhA1: Active Enhancer 1,

10_EnhA2: Active Enhancer 2, 11_EnhWk: Weak Enhancer, 12_ZNF/Rpts: ZNF genes & repeats,

13_Het: Heterochromatin, 14_TssBiv: Bivalent/Poised TSS, 15_EnhBiv: Bivalent Enhancer,

16_ReprPC: Repressed PolyComb, 17_ReprPCWk: Weak Repressed PolyComb, 18_Quies: Quiescent/Low

Figure S10

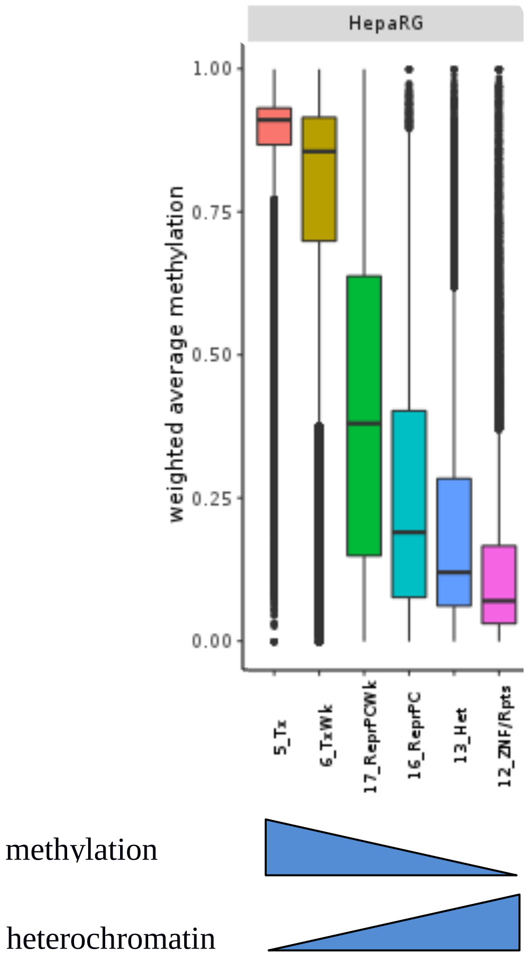

Figure S10. DNA methylation erosion is accompanied by increasing of heterochromatic marks. Methylation levels across selected ChromHMM segments showing that DNA-demthylation is accompanied by heterochromatization in HepaRG. labels are the following:

5_Tx: Strong transcription, 6_TxWk: Weak transcription, 12_ZNF/Rpts: ZNF genes & repeats,

13_Het: Heterochromatin, 16_ReprPC: Repressed PolyComb, 17_ReprPCWk: Weak Repressed PolyComb

Figure S11

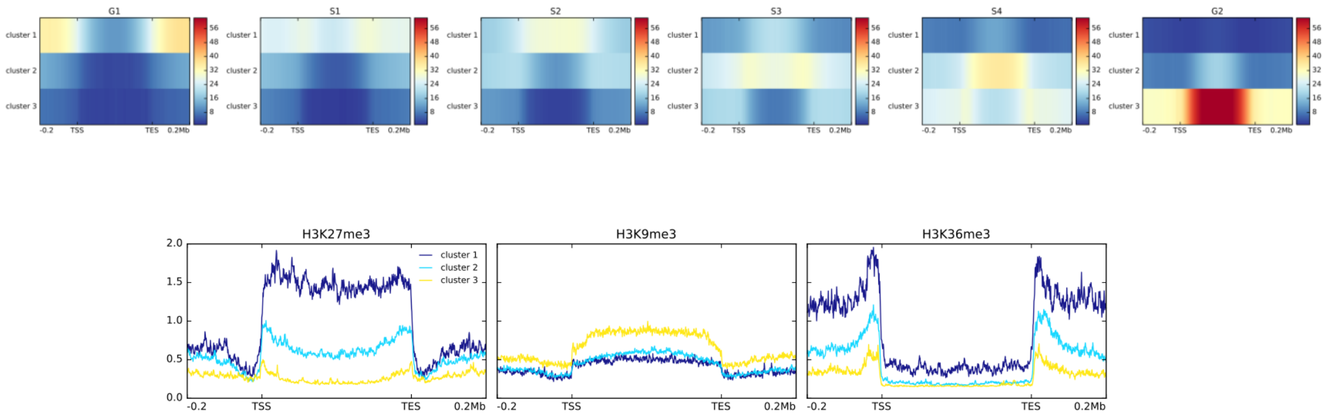

Figure S11. PMDs and heterochromatic marks demarcate distinct domains of late DNA-replication. Heterochromatization at PMDs during cell cycle in IMR90 (related to Figure 4A, B of HepG2)

Figure S12

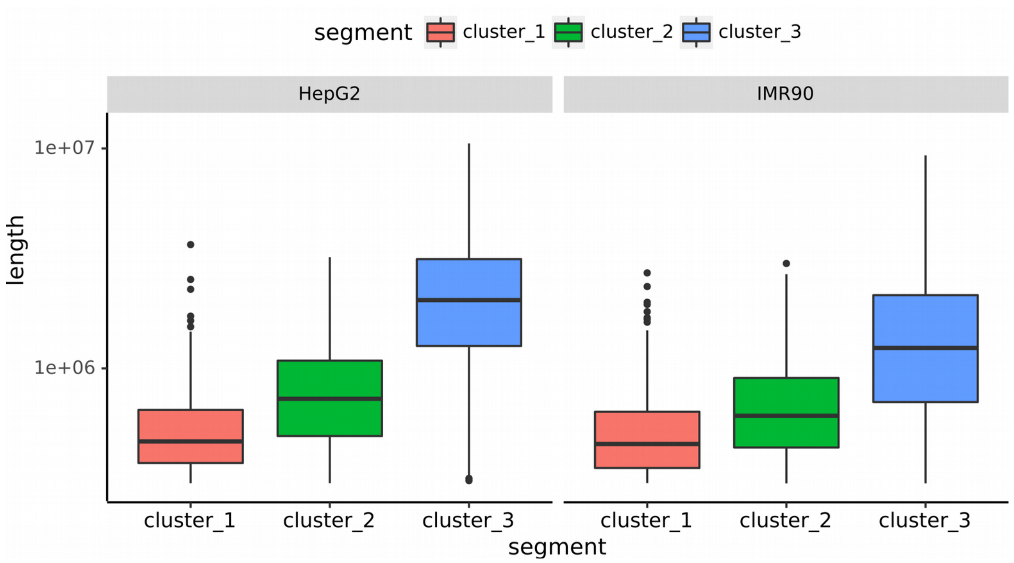

Figure S12. Cluster lengths

Figure S13

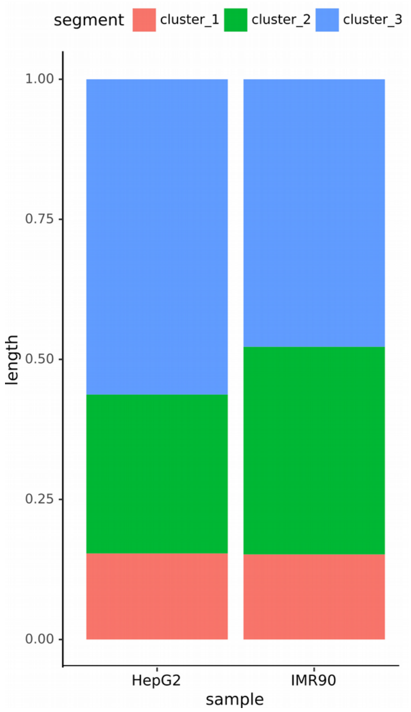

Figure S13. Clusters percentage of the genome

Figure S14

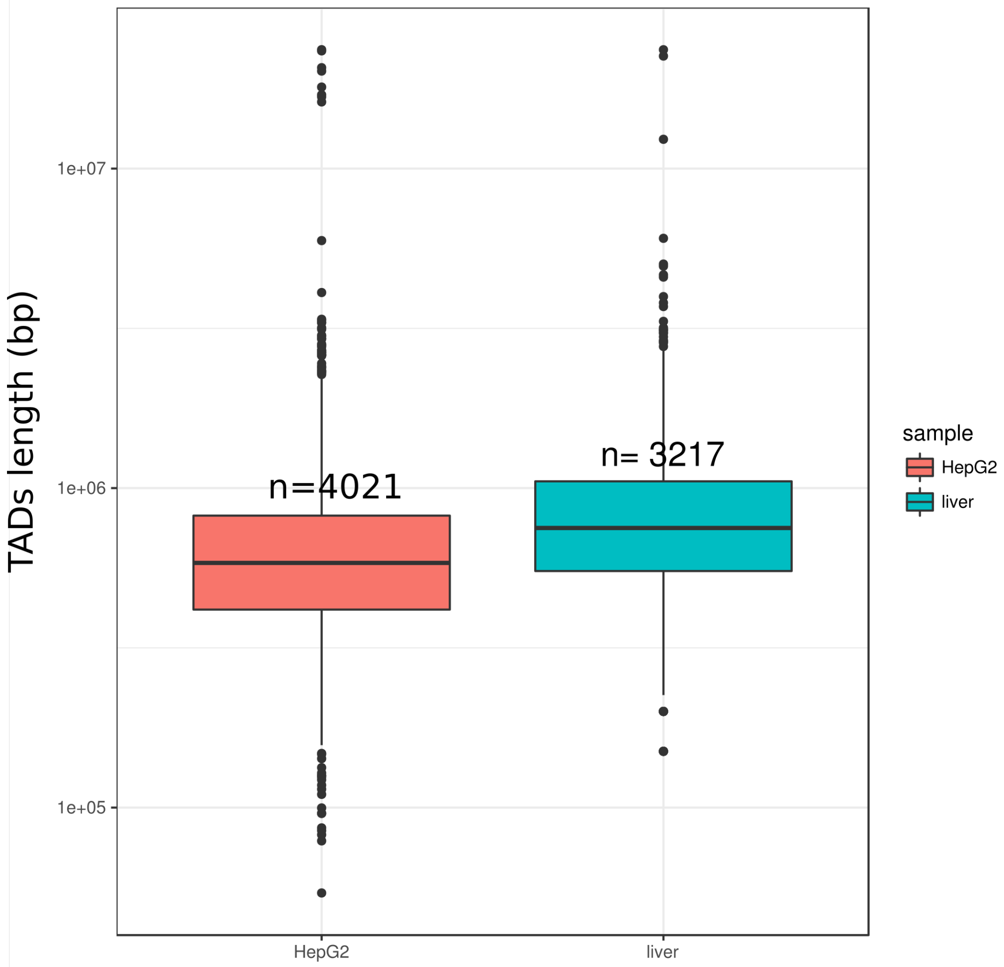

Figure S14. TAD lengths. TAD lengths in log10 scale for HepG2 and liver. The number above each boxplot represent the number of TADs for the corresponding sample.

Figure S15

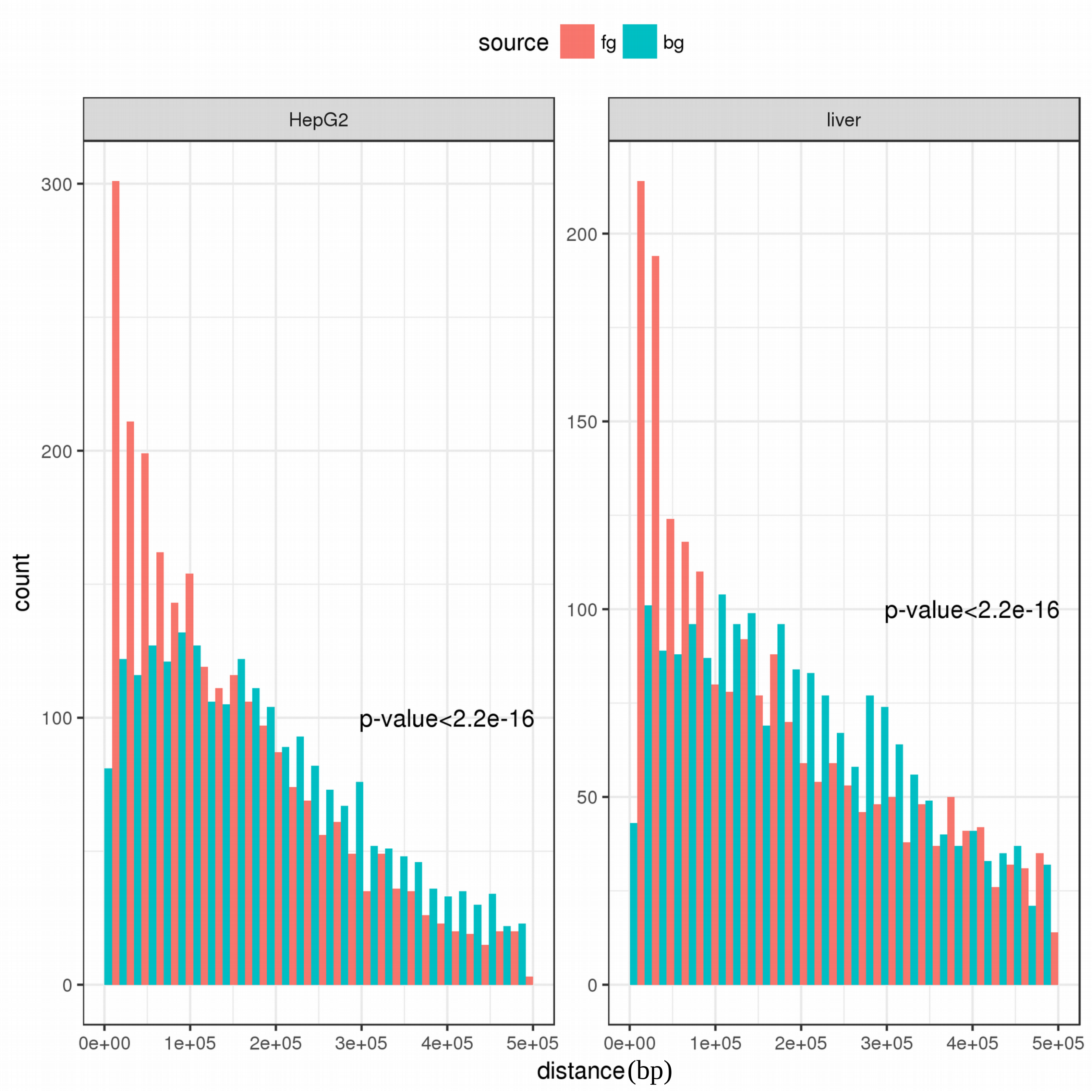

Figure S15. Distance distribution between TAD borders and PMD borders. TAD borders are closer to PMD borders than randomized set of borders according to Wilcoxon test (p-value < 2.2e-16). Fg (in red) represent the distance distribution between TAD borders and PMD borders. Bg (in green) represent the distance distribution between TAD borders and randomized test borders.

Figure S16

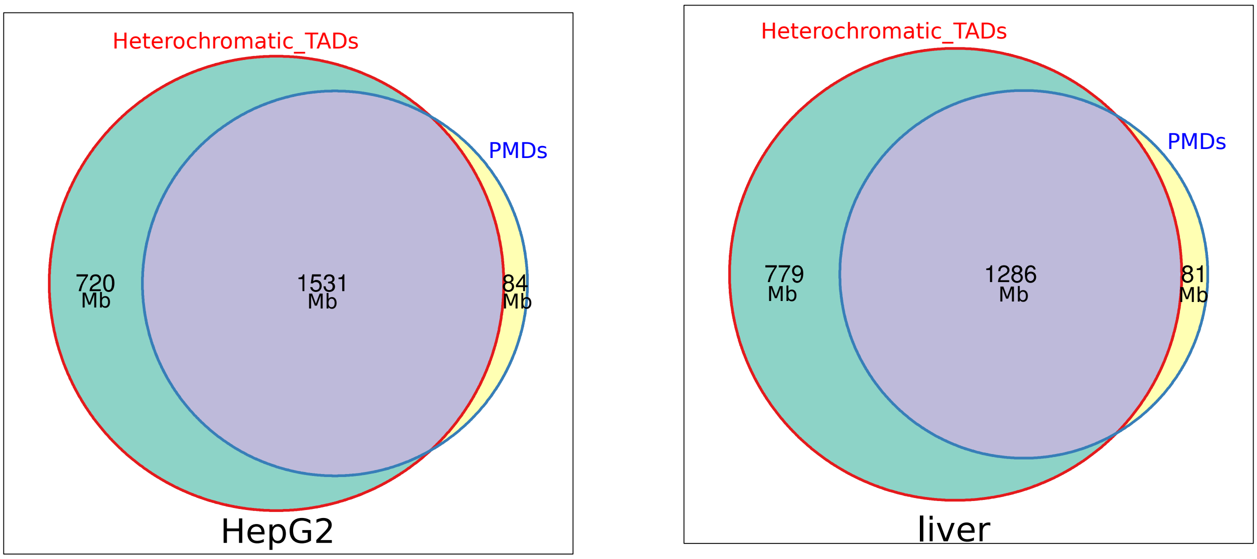

Figure S16. PMDs and heterochromatic TADs overlap. 94% of PMDs overlap with heterochromatic TADs in HepG2 and liver. Heterochromatic TADs in HepG2 form ~ 2.25 Gb of the genome while they are less in liver ~ 2.0 Gb.

Figure S17

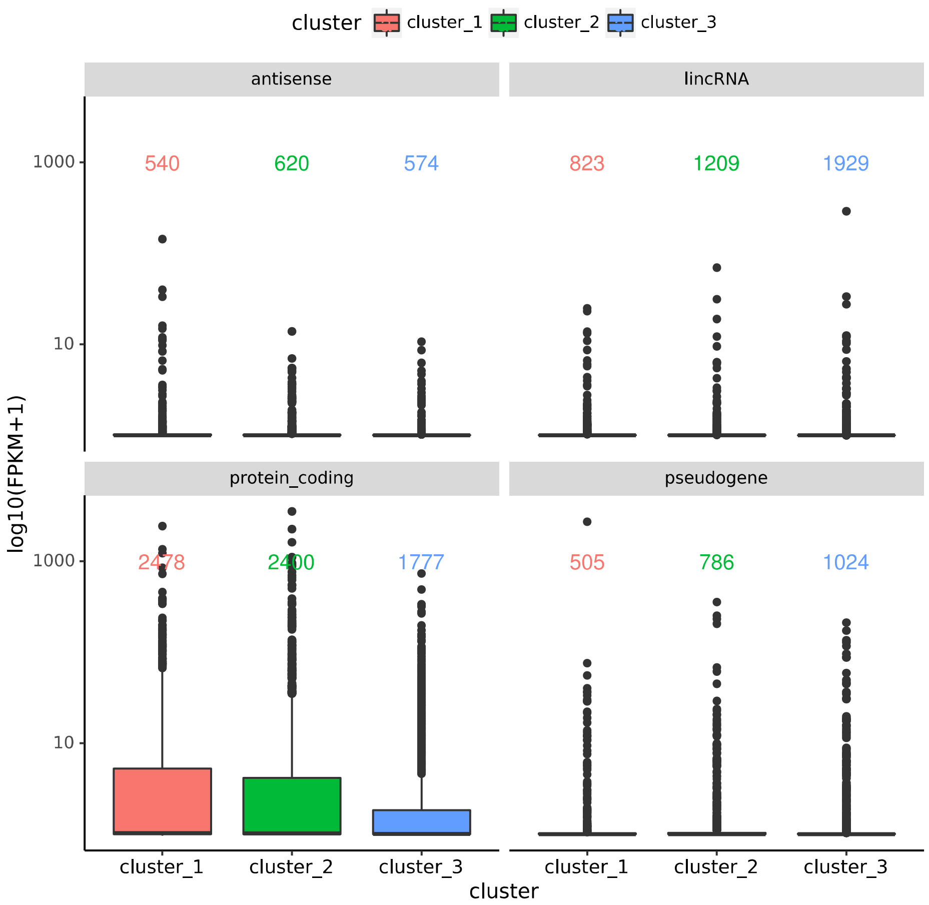

Figure S17. Number of the genes and FPKM values in some gene classes in each cluster

Figure S18

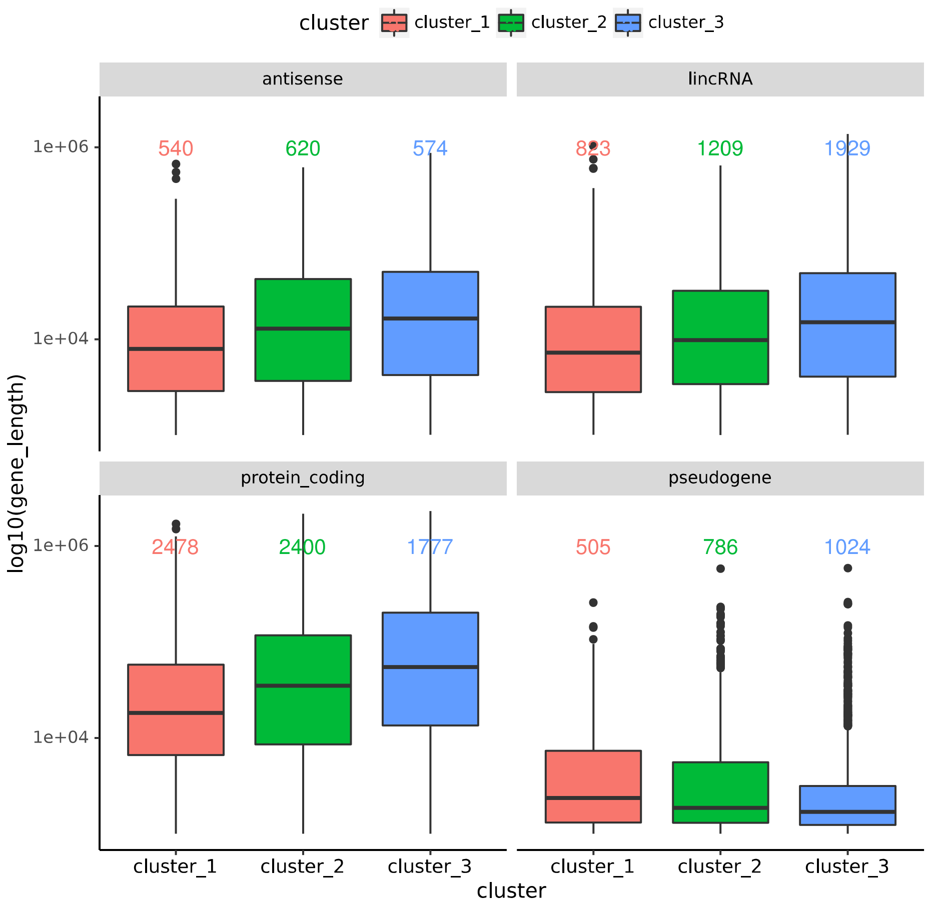

Figure S18. Gene lengths in some gene classes in each cluster

Figure S19

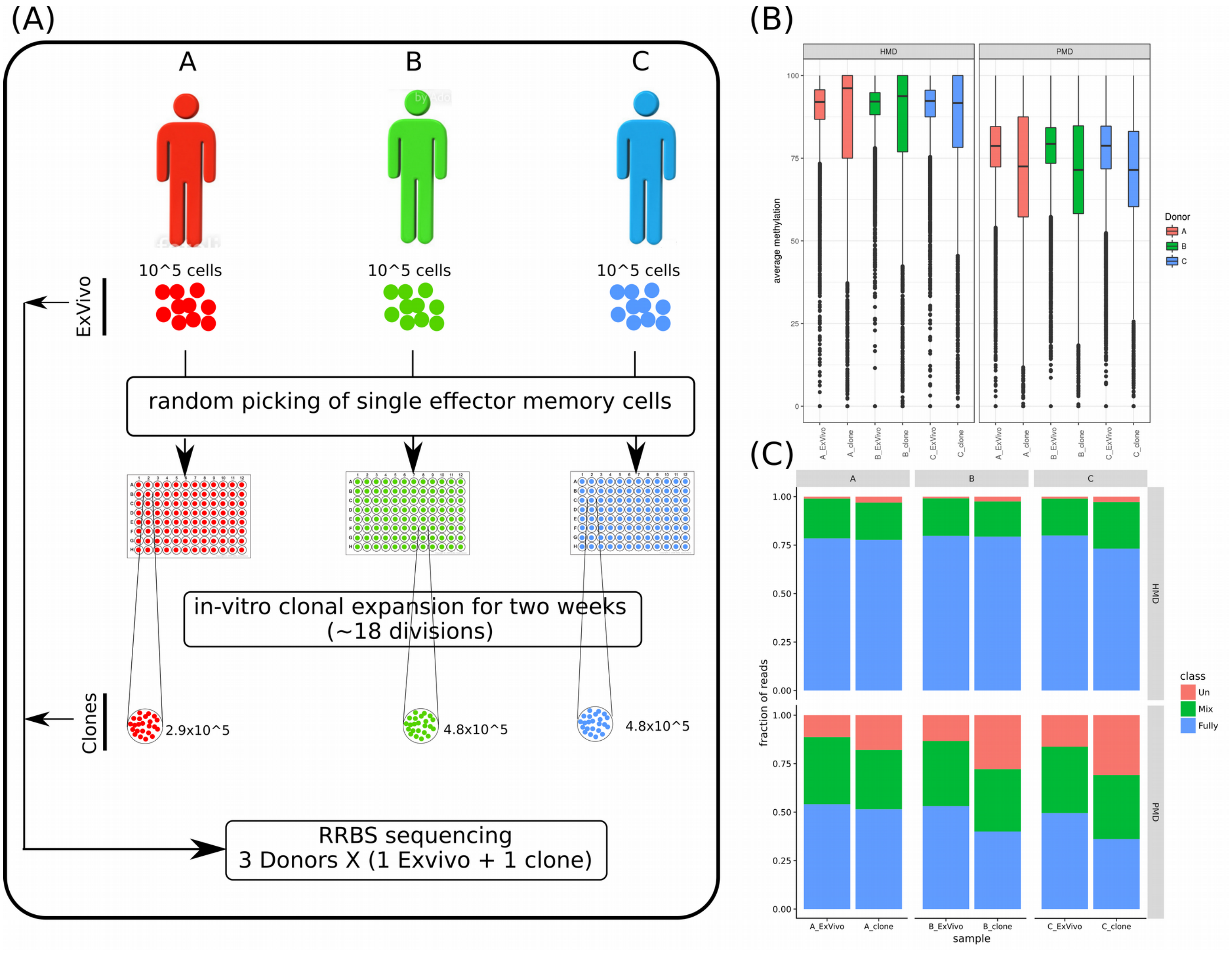

Figure S19: Clonal cultures of T memory cells. (A) Workflow of the designed experiment for T memory single cell expansion (details in the supplementary materials). (B) Average methylation across the three donors (ex vivo + in vitro cloned single cells) in PMDs/HMDs (as defined by TEM sample in Durek et al). A clear loss of methylation in PMDs of in vitro samples was obsereved. (C) CpG pattern class distributions in the PMDs/HMDs/others (as described in the supplementary materials). The fraction of mixed patterns (green), in PMDs, does not change due to the expansion process. The fraction of fully methylated patterns (blue) decreases and is compensated by an increase in the unmethylated patterns (red).

Figure S20

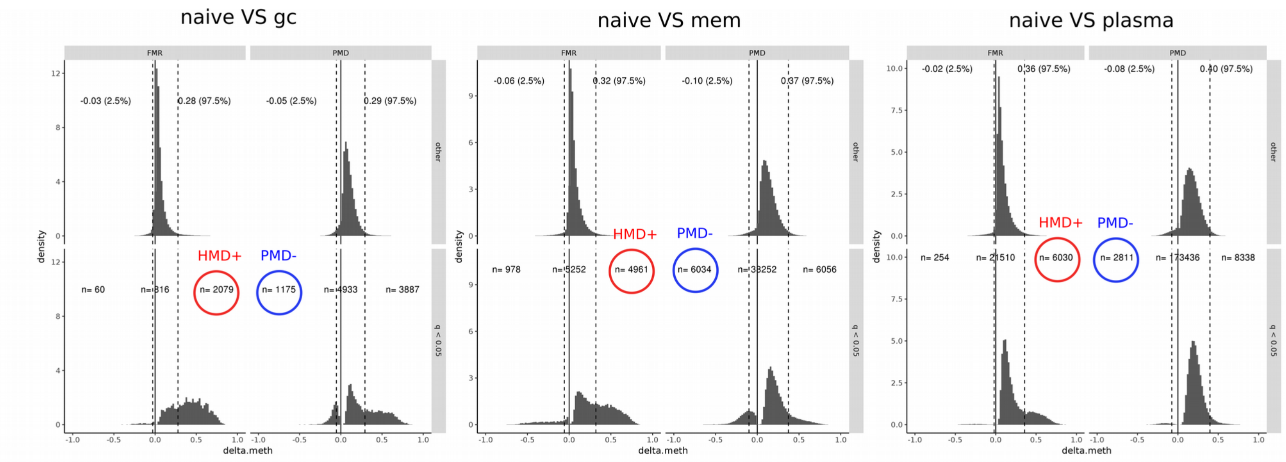

		PMD+	PMD-	HMD+	HMD-
naive_VS_gc		3887	1175	2079	60
naive_VS_mem		6056	6034	4961	978
naive_VS_plasma	adaptive filtering (AF)	8338	2811	6030	254
	w/o adaptive filtering	28014	95	6650	82
gc_VS_mem		123	1320	156	243
gc_VS_plasma		47	237	18	23
mem_VS_plasma		108	44	85	83

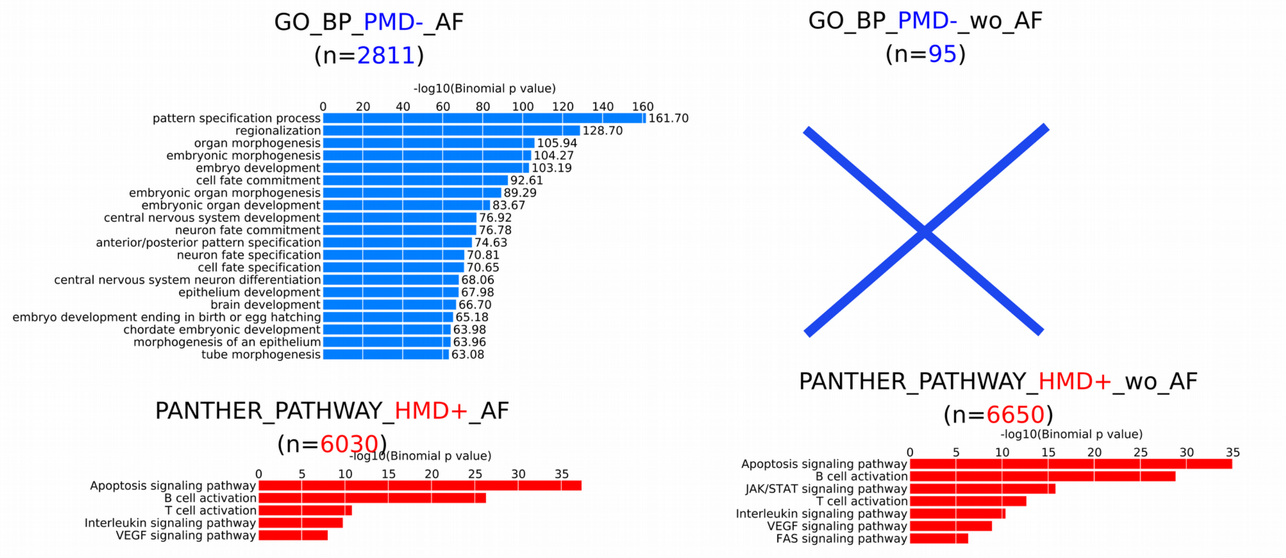

Figure S20. Adaptive filtering. DMRs analysis of B-cells during differentiation using the adaptive filtering method from Durek et al 2016. naive=Naive B-cells, gc=germinal center B-cells, mem= memory B-cells. Significant DMRs are annotated as HMDs+/- or PMDs+/-. Gene enrichment analysis for HMD+ and PMD- was performed using GREAT tool.

Supplementary methods

Clonal cultures of T memory cells

CD4+ T memory cells (CD3+ CD4+ CD45RA- CD45RO+ CD25-) from three different donors were sorted by flow-cytometry either as a bulk culture ('ex vivo' sample) or in a single-cell format into 96 well-plates. Single cells were cultured in the presence of a TCR stimulus (human T Cell Activation/Expansion Kit, Miltenyi Biotech) and human interleukin-2. After expansion, single clonal cultures were picked and treated with bisulfite for RRBS analysis. All three ex vivo and the matching three clones were sequenced.

CpG patterns analysis:

We considered four consecutive CpGs to be in the same read and classified the patterns into three classes; fully methylated patterns (Fully), fully unmethylated patterns (Un) and the remaining pattern combinations are “mix”. We calculated the fraction of each pattern genome wide in each ex vivo and the matched cloned sample. The patterns were stratified across HMDs and PMDs as defined from Figure 3A in the MS. We considered cluster 1 as “HMDs”, cluster 5 as “PMDs” and the remaining clusters as “others”.

References

Durek, Pawel, et al. "Epigenomic profiling of human CD4+ T cells supports a linear differentiation model and highlights molecular regulators of memory development." Immunity 45.5 (2016): 1148 1161.
