# Supplementary material for: A comprehensive analysis of 195 DNA methylomes reveals shared and cell-specific features of partially methylated domains
Source: Genome Biol. 2018 Sep 28;19:150. doi: 10.1186/s13059-018-1510-5 (PMC6161375; doi:10.1186/s13059-018-1510-5)
Supplement: Supplementary file 3 — Review history. (DOCX 54 kb) [file 13059_2018_1510_MOESM3_ESM.docx]

Review History

**First round of review**

**Reviewer 1**

**Are the methods appropriate to the aims of the study, are they well described, and are necessary controls included?**
Yes, the Methods are appropriately described

**Are the conclusions adequately supported by the data shown?**

No, the conclusions about demethylation in the formation of PMDs are not supported by the data, as described in detail in the comments to authors.

**Are sufficient details provided to allow replication and comparison with related analyses that may have been performed?**

It was unclear if the ChromH3M code, tracks and PMD calls will be publicly available through the IHEC or other site for others to utilize

**Does the work represent a significant advance over previously published studies? Please explain.**

As mentioned below, the title does not state a novel finding and the innovation is not so much is what insights they found, but from the weight of evidence of analyzing PMDs from 171 human WGBS datasets, as well as in the tool they used to perform this large-scale analysis.

**Is the paper of broad interest to others in the field, or of outstanding interest to a broad audience of biologists?**

Yes: The study of PMDs has been a small but emerging subset of papers, but all biologists should be made more aware of their important signature of chromatin state and tissue identity. Therefore, because of its size, this study could help make PMDs more in the mainstream genomic vernacular.

Comments to authors:

This is a potentially important bioinformatics study that examined coverage and features of partially methylated domains (PMDs) across 171 human and 24 mouse WGBS data sets from public repositories. The conclusions made about PMDs have been previously observed by prior smaller studies examining PMDs, including their cell type specificity and coincidence with heterochromatic late replicating regions. However, this study introduces a new tool ChromH3M for meta-analyses of methylation and chromatin states and provides an important large body of evidence supporting PMDs as important and biologically meaningful entities in the mammalian genome. The manuscript as written, has several flaws in the interpretation of PMDs as "demethylated" and the title, abstract, and text would need to be rewritten to present a less biased interpretation of the evidence or new data demonstrating direct evidence supporting demethylation in PMD formation at the single cell level.

Major concerns

1.	The major concern is with the authors interpretation throughout the abstract and text of demethylation as an explanation for the PMD state in tissues. They do not consider the alternative explanation that the appearance of PMD gains during proliferation and/or differentiation of a tissue or cell type may be due to clonal selection of cells that had PMDs in the prior population, but in numbers too low to be detected in the population. Single cell methylation sequencing technologies, such as the approach recently published from Swift Biosciences (Luo et al, Science, 2017) would be required to demonstrate the conclusion of demethylation rather than selection. Alternatively, the authors should remove all conclusions about demethylation or decreased methylation as an active process in the creation of PMDs from the abstract and text of the manuscript and instead have a section the Discussion dedicated to the alternative hypotheses about formation or inheritance of PMDs states and how they could be distinguished by future studies.

2.	The PMDs detected in non-transformed cell types such as PHH shown if Fig. 1 have a much less dramatic drop in methylation levels over PMDs than transformed cell types such as HepG2 cells. Calling of PMDs in past publications has been in tissue types where these dramatic drops have been impossible to ignore, such as tumors, transformed cell lines, placenta, oocytes, and early embryos. It is interesting that the authors are now detecting PMDs by sensitive HMM approaches in tissues where there is a more subtle but detectable drop over regions that have been previously defined as cell type specific PMDs in transformed cells of the same lineage (such as HepG2 in Figure 1). While I think it is still appropriate for the authors to use the term "PMD" to describe and call these more subtle regions of lower methylation, it is important that they consider the distinction between the two type of PMDs they show in Fig 1 and discuss in their interpretations possible explanations, such as a subset of cells containing large drop PMDs within the tissue showing the more subtle drops in methylation levels.

3.	The title as stated is not a novel finding for the field, as it has been shown by multiple prior studies on PMDs cited by the authors that PMDs are cell type specific features able to define cell lineage. What is new about the current study is the weight of evidence that comes from calling PMDs in 171 WGBS methylomes in support of the prior observations and the insights into chromatin, replication, and differentiation gained by integration with ChIP-seq, HiC-seq, Repli-seq, and RNA-seq datasets. The ChromH3M is also somewhat novel, although it basically is a tool built from prior similar tools. The title should be revised to accurately reflect the novelty of this study to the field.

4.	The authors should use the previously established term HMD for "highly methylated domain" rather the FMR for "fully methylation region" because these regions are not truly fully methylated to 100% and "domain" is conventionally larger than "region" and these areas cover large Mb wide domains in the genome. Also, UMR for "unmethylated region" seems also to be a misnomer since the UMR marked regions shown in Figure 3A in HepG2 and HepaRG cells have clearly detectable methylation. It is important for consensus in the field and accuracy of the labels, that these terms are named appropriately.

5.	While the abstract mentions that both human and mouse methylomes were analyzed, there is almost no mention of the 24 mouse tissues examined for PMDs except for a sentence on p. 6 and Suppl. Fig. S7. In the Discussion, the authors use the term "conserved PMD" but it appears to refer to PMDs conserved between cell types, not those conserved between mouse and human. There should be some attempt made to examine overlapping syntenic regions between mouse and human for PMD coverage in similar tissues. Also, the term "conserved PMDs" should be used for evolutionary conservation while a different term, such as "All PMDs" should be used for PMDs that are consistent across all tissues.

Minor concerns

1.	It would be useful in the Introduction to clearly state that PMDs are defined not by an absolute threshold or level of % methylation, but by a HMM-based detection of a state of relative lower methylation compared to neighboring regions of the genome.

2.	The references throughout the entire text are represented a [?] rather than numbers, which may be a problem with conversion, but made it hard to tell the specific citations.

3.	The first sentence of the Results section of the abstract "PMDs are ubiquitous signatures covering up to 75% of the genome…" should be replaced by "PMDs are ubiquitous signatures that can be detected in up to 75% of the genome…" to reflect the cellular heterogeneity aspect of the nature of PMDs mentioned above.

4.	PHH cell are not defined in the legend of Figure 1.

5.	To make the figures more self-explanatory, use actual names for the classification, like "early/mid S phase, mid/late S phase, and very late S phase" instead of Cluster 1,2,3. This is one example but there are several examples where the figures can be labelled better to improve clarity, including the three clusters defined in Figure 3C.

6.	The authors should discuss the findings of PMDs in oocytes and preimplantation embryos and how that relates to the biological implications of PMDs in cells and tissues of the adult.

7.	In the Discussion, the suggestion of PMDs reflecting constituative heterochromatin is not consistent with the cell type specificity of their patterns and should be removed or reconsidered.

8.	In the Discussion, p. 12, lines 23-25, the reference seems to be quoted incorrectly. The conclusion from that paper was that A/B chromatin compartments were formed independently of DNA methylation in myocytes.

9.	The abstract states 190 WGBS, but 171 human plus 24 mouse is 195.

**Reviewer 2**

**Are the methods appropriate to the aims of the study, are they well described, and are necessary controls included?**
Yes the methods are appropriate to the aims of the study. There are a few minor comments that need addressing such as detailing whether smoothing of methylation values was used and if so across how many CpGs.

**Are the conclusions adequately supported by the data shown?**

Yes for the most part. If the reviews can address my major comments.

**Are sufficient details provided to allow replication and comparison with related analyses that may have been performed?**

Yes

**Does the work represent a significant advance over previously published studies? Please explain.**

This represents the first time in which analysis has be performed on a large number of WGBS to assess the differences in PMDs across cell types. This has revealed that PMDs are cell type specific and that they are linked with heterochromatic signatures. This is a significant advancement over previously published papers.

**Is the paper of broad interest to others in the field, or of outstanding interest to a broad audience of biologists?**

No

Comments to author

The paper by Salhab et al. investigates a large cohort of WGBS data and analyses the data using a segmentation tool to break up the genome into 4 types: FMRs, PMDs, LMRS, UMRs. They discover that PMDs cover 50-75% of all epigenomes yet are highly cell specific with only 26% of PMDs shared among all data analysed. The analysis is strong but I have a few comments that I feel should be addressed.

Major Comments:

1. One potential concern is that PMD identification is dependent on the quality of the data, and in particular the coverage of the WGBS data. Could you show whether there is any correlation between coverage of dataset and amount of PMDs obtained. Also are PMDs in each individual sample obtained at regions with lower coverage or is the distribution of coverage of PMDs similar to that of random regions.

2. The authors also reveal that PMDs may have distinct heterochromatic signatures that is related to replication timing. I therefore wonder whether they think it is possible that some of the cell type differences that they see are differences in the distribution of cell cycle stages in the samples?

3. The TAD analysis is very weak. For example: "we find that several TADs may overlap with one PMD. Still many PMD boundaries overlap with (strong) TAD boundaries (30%)" Can you test whether this is significant? How did you define this overlap?

Minor Comments:

1. Can you provide mapping statistics and coverage statistics for each dataset.

2. Did you perform any smoothing of methylation across CpGs?

3. Figure 1 is not referenced in the manuscript.

4. P8 Line 2 and line 3 Seem to say the same thing twice

5. There seems to be a red rectangle on Figure 4c covering most of the data.

Authors Response

Point-by-point responses to the reviewers’ comments:

Reviewer #1: This is a potentially important bioinformatics study that examined coverage and features of partially methylated domains (PMDs) across 171 human and 24 mouse WGBS data sets from public repositories. The conclusions made about PMDs have been previously observed by prior smaller studies examining PMDs, including their cell type specificity and coincidence with heterochromatic late replicating regions. However, this study introduces a new tool ChromH3M for meta-analyses of methylation and chromatin states and provides an important large body of evidence supporting PMDs as important and biologically meaningful entities in the mammalian genome. The manuscript as written, has several flaws in the interpretation of PMDs as "demethylated" and the title, abstract, and text would need to be rewritten to present a less biased interpretation of the evidence or new data demonstrating direct evidence supporting demethylation in PMD formation at the single cell level.

Major concerns

1. The major concern is with the authors interpretation throughout the abstract and text of demethylation as an explanation for the PMD state in tissues. They do not consider the alternative explanation that the appearance of PMD gains during proliferation and/or differentiation of a tissue or cell type may be due to clonal selection of cells that had PMDs in the prior population, but in numbers too low to be detected in the population. Single cell methylation sequencing technologies, such as the approach recently published from Swift Biosciences (Luo et al, Science, 2017) would be required to demonstrate the conclusion of demethylation rather than selection. Alternatively, the authors should remove all conclusions about demethylation or decreased methylation as an active process in the creation of PMDs from the abstract and text of the manuscript and instead have a section the Discussion dedicated to the alternative hypotheses about formation or inheritance of PMDs states and how they could be distinguished by future studies.

We thank the reviewer for raising this important point. It is indeed very difficult to distinguish between a consecutive/progressive loss of DNA-methylation (demethylation per molecule) in all cells and a clonal selection/expansion of subpopulations of preexisting partially methylated chromosomes (cell subpopulations). The generation of single cell methylomes (as described by Luo et al, 2017 and others) might in fact allow to address this question. However unfortunately single cells datasets do not reach a genome wide coverage compatible with comprehensive and comparative PMD analyses. Conventional WGBS (most datasets used had coverage >30x) may indeed only represent a proportion of the true methylation variation. To approach this question more systematically we performed additional analyses and calculated the entropy-like score as introduced by Landan et al. 2012. Indeed the epipolymorphism scores of PMDs are distinctively higher than expected for bimodal populations (Figure S19). We added the analysis for T-cells as a new supplementary figure and amended the text in our discussion accordingly (p11, line 33 & p12, lines 1-11). Still we admit that even an increased epipolymorphism score does not formally exclude that an enhanced mosaicism in PMDs may not originate from demethylation but rather a clonal expansion of chromosomes with lower methylation levels in PMDs. In our previous work, Durek et.al 2016, we found that PMDs become more pronounced in memory T- and B- cells. Here we show in addition that the entropy scores increase. We suggested that for these cells the increased entropy might be linked to cell proliferation and imperfect maintenance during cell divisions rather than clonal selection. Similar scenarios have been proposed for ESC caused by the lack of Uhrf1 (von Meyenn et al. 2016). In other cases (of not dividing or slow growing cells) this may not be the case. In our revised text we mention both possibilities.

2. The PMDs detected in non-transformed cell types such as PHH shown if Fig. 1 have a much less dramatic drop in methylation levels over PMDs than transformed cell types such as HepG2 cells. Calling of PMDs in past publications has been in tissue types where these dramatic drops have been impossible to ignore, such as tumors, transformed cell lines, placenta, oocytes, and early embryos. It is interesting that the authors are now detecting PMDs by sensitive HMM approaches in tissues where there is a more subtle but detectable drop over regions that have been previously defined as cell type specific PMDs in transformed cells of the same lineage (such as HepG2 in Figure 1). While I think it is still appropriate for the authors to use the term "PMD" to describe and call these more subtle regions of lower methylation, it is important that they consider the distinction between the two type of PMDs they show in Fig 1 and discuss in their interpretations possible explanations, such as a subset of cells containing large drop PMDs within the tissue showing the more subtle drops in methylation levels.

We appreciate the criticial comments on distinct PMD structures in primary cells, tissues and cell culture samples, respectively. Indeed all PMD tracks of primary cells and tissues (Fig. 1) used for the integrative analysis (171 human and 24 mouse samples excluding cell lines) were generated with MethylSeekR using the same parameter settings (see modified method section “MethylSeekR segmentation”) while for cell culture cells (such as HepG2 and HepaRG, p. 8, line 24) we had to use a two-state HMM to cope with the extreme almost bimodal structure in HepG2. This distinct approaches are now clearly marked in the text.

In all comparisons with HepG2 the term PMD was maintained to physically link true PMDs identified in primary cells to corresponding “PMD like” regions in these cancerous cell lines despite of their very distinct low methylation status (almost UMRs). We believe that the increased loss of PMD methylation in cultured cancer cells (such as HepG2 and HepaRG) in comparison to primary hepatocytes are likely to be a general consequence of cell culturing and progressive proliferation.

3.The title as stated is not a novel finding for the field, as it has been shown by multiple prior studies on PMDs cited by the authors that PMDs are cell type specific features able to define cell lineage. What is new about the current study is the weight of evidence that comes from calling PMDs in 171 WGBS methylomes in support of the prior observations and the insights into chromatin, replication, and differentiation gained by integration with ChIP-seq, HiC-seq, Repli-seq, and RNA-seq datasets. The ChromH3M is also somewhat novel, although it basically is a tool built from prior similar tools. The title should be revised to accurately reflect the novelty of this study to the field.

We agree that PMDs were reported as cell type specific features. However, prior studies were primarily based on cancer tissues and cultured cell lines with low number of primary tissues. In addition some of them are based on low coverage 450k array data and not genome wide WGBS. Here our analysis is indeed much deeper and cover a large spectrum of human cell types (157 primary cells + 14 tissues) and 24 mouse tissues. To make this more clear we modified the title as suggested:

“A comprehensive analysis of 195 DNA-methylomes reveals shared and cell specific features of partially methylated domains”

4.The authors should use the previously established term HMD for "highly methylated domain" rather the FMR for "fully methylation region" because these regions are not truly fully methylated to 100% and "domain" is conventionally larger than "region" and these areas cover large Mb wide domains in the genome. Also, UMR for "unmethylated region" seems also to be a misnomer since the UMR marked regions shown in Figure 3A in HepG2 and HepaRG cells have clearly detectable methylation. It is important for consensus in the field and accuracy of the labels, that these terms are named appropriately.

We used the term “FMRs” for historical reasons as it has been used in previous studies Stadler et.al 2011, Burger et al. 2013, Durek et al. 2016, Nothjunge et al. 2017, Gilsbach et al. 2018. However we admit that the reviewer raises an important point and we take up the suggestions to systematically replace the terms “FMR” by “HMD” in both text and figures. HMD indeed better reflects the intention of our domain focused analysis.

The use of the term UMRs for our analysis is indeed more complex. According to Burger et.al 2013 (MethylSeekR) UMRs are defined as regions with methylation below 50% and at least 30 CpGs (p.3, lines 19-22). While in all cells this definition results in the calling of short and discrete UMRs, additional new extended UMRs appear only in the cell lines HepG2 and HepaRG as domain like structures. These new UMRs mostly overlap with PMDs of primary hepatocytes (p.8 , line 18-20). To avoid the introduction of a new nomenclatures we keep up with the established term (UMR) across all samples (in Figure 3A).

5. While the abstract mentions that both human and mouse methylomes were analyzed, there is almost no mention of the 24 mouse tissues examined for PMDs except for a sentence on p. 6 and Suppl. Fig. S7. In the Discussion, the authors use the term "conserved PMD" but it appears to refer to PMDs conserved between cell types, not those conserved between mouse and human. There should be some attempt made to examine overlapping syntenic regions between mouse and human for PMD coverage in similar tissues. Also, the term "conserved PMDs" should be used for evolutionary conservation while a different term, such as "All PMDs" should be used for PMDs that are consistent across all tissues.

- Our ChromH3M analysis of mouse samples shows its applicability for other non –human mammalian species. The question of an evolutionary conservation of PMDs is of course very interesting and important and we carefully reexamined the possibility to address this. We realized that a comprehensive genome wide analysis of synthenic regions is very much dependent on parameter settings introducing additional variables not easy to control. Second currently the publically available high quality (coverage) WGBS data sets for mouse do not really overlap with matching cell types or tissues from human. We therefore refrained from addressing this interesting question.

To avoid any possible confusion between PMDs shared between cell types and PMDs conserved between species (i.e. in the same cell type) we replace the term “conserved PMDs” by “shared PMDs”. Shared PMDs defines PMDs that are shared between different cell types in the same species.

- C- PMDs was replaced by shared-PMDs in Figure 2B

- C- FMRs was replaced by shared-HMDs in Figure 2B

- the text changed accordingly (p.6, line 4 & p.10, lines 12-13 & p.11, line 14)

Minor concerns

1. It would be useful in the Introduction to clearly state that PMDs are defined not by an absolute threshold or level of % methylation, but by a HMM-based detection of a state of relative lower methylation compared to neighboring regions of the genome.

- it’s already mentioned in p3,line 14-18 in the old MS and we state it again in “Background” section (p3, lines 11-13 in the new MS)

2. The references throughout the entire text are represented a [?] rather than numbers, which may be a problem with conversion, but made it hard to tell the specific citations.

- We solved this issue during the re-submission process.

3. The first sentence of the Results section of the abstract "PMDs are ubiquitous signatures covering up to 75% of the genome…" should be replaced by "PMDs are ubiquitous signatures that can be detected in up to 75% of the genome…" to reflect the cellular heterogeneity aspect of the nature of PMDs mentioned above.

- We changed it as suggested.

4. PHH cell are not defined in the legend of Figure 1.

- “hepatocytes” was replaced by “primary human hepatocytes” in Figure1’s legend

5. To make the figures more self-explanatory, use actual names for the classification, like "early/mid S phase, mid/late S phase, and very late S phase" instead of Cluster 1,2,3. This is one example but there are several examples where the figures can be labelled better to improve clarity, including the three clusters defined in Figure 3C.

- clusters have been renamed into cluster 1(early/mid S), cluster 2 (mid/late S) and cluster 3 (very late S/G2) in Figure 4 and in the text accordingly (p.9 lines 1-6).

6. The authors should discuss the findings of PMDs in oocytes and preimplantation embryos and how that relates to the biological implications of PMDs in cells and tissues of the adult.

- We are grateful for raising this point. We now include a small discussion on the points raised by Schroeder et al. 2015 (p.12, lines 12-18).

7. In the Discussion, the suggestion of PMDs reflecting constituative heterochromatin is not consistent with the cell type specificity of their patterns and should be removed or reconsidered.

- We distinguished between short PMDs and long PMDs in HepG2. We hypothesized that the former is facultative heterochromatin (more flexible) while the latter is constitutive heterochromatin (much more compact) (p.10, lines 32,33 & p.11, line 1)

8. In the Discussion, p. 12, lines 23-25, the reference seems to be quoted incorrectly. The conclusion from that paper was that A/B chromatin compartments were formed independently of DNA methylation in myocytes.

- We disagree and do not see an incorrect quotation of the Nothjunge’s paper. Our interpretation is based on Figure2a,b from Nothjunge et al. 2017 to which the authors state: “A/B compartments precede manifestation of DNA methylation”. Moreover in their figure legend they state: “DNA methylation and gene expression succeeds formation of A/B compartments”. Based on these statements and our (matching) observations we speculate in the discussion about a temporal link of heterochromatic B compartments and PMDs.

9. The abstract states 190 WGBS, but 171 human plus 24 mouse is 195.

- “more than 190” was changed to “171 and 24 ” (p.2,lines 5)

- “more than 190” was changed to “195” (p.13, line 24)

Reviewer #2: The paper by Salhab et al. investigates a large cohort of WGBS data and analyses the data using a segmentation tool to break up the genome into 4 types: FMRs, PMDs, LMRS, UMRs. They discover that PMDs cover 50-75% of all epigenomes yet are highly cell specific with only 26% of PMDs shared among all data analysed. The analysis is strong but I have a few comments that I feel should be addressed.

Major Comments:

1. One potential concern is that PMD identification is dependent on the quality of the data, and in particular the coverage of the WGBS data. Could you show whether there is any correlation between coverage of dataset and amount of PMDs obtained. Also are PMDs in each individual sample obtained at regions with lower coverage or is the distribution of coverage of PMDs similar to that of random regions.

- It is of course true that PMD identification is dependent on the data quality. We used MethylSeekR (Burger et al 2013) to identify PMDs and followed the recommendations. We now added coverage statistics to document this in the additional file 1 (related to minor comment 1). We could not find any correlation or obvious relation between average coverage and the amount of PMDs obtained (see figure below). Moreover in our ChromH3M analysis cells from the same origin cluster together, regardless of the consortium that has produced and processed them (not shown). As an example, the lowest coverage TCD4 samples from Blueprint (coverage ~ 7X) cluster together with the TCD4 samples from DEEP (mean coverage ~ 21X).

**Second round of review**

**Reviewer 1**

The authors have made a reasonable attempt to respond to reviewer critiques, although mostly with wording changes and written responses rather than new experimental or comprehensive bioinformatic analyses. My prior concerns about lack of novelty of the studies analyses and findings still stand.

Unfortunately for the authors, a new publication on a very comprehensive analysis of PMDs across a larger sampling of WGBS data sets was recently published and is attached. Therefore the author's claim in the abstract "Here, we present a first comprehensive comparative analysis of PMDs across 171 and 24 WGBS methylomes of human and mouse cells, respectively..." is no longer appropriate after the Zhou et al publication.

What could be novel about the current study, if the authors are willing to address the question head on and aggressively is the major unanswered question in the field: is the gain of PMDs with mitotic cell divisions due to demethylation of all the cells or clonal selection of a smaller subset of cells that already contain PMDs. The Zhou et al manuscript, while it did other analyses quite elegantly, was not able to answer this question, leading to the rather squishy wording in the title that PMDs are "linked" to mitotic cell division (rather than caused or associated). The authors attempt to answer my concern about this outstanding question, but the analysis was unsatisfying. While the "epipolymorphism score" appears to be a reasonable approach, the model of a simple bimodal population of completely methylated and completely unmethylated molecules is not what is expected in an actual situation where a cell population containing <1% of cells containing PMDs (so apparently PMD-) expands to 10%, 20%, 50%, etc of the cell population through clonal expansion, resulting in a PMD+ classification of the cell population. These cell population shifts could be bioinformatically modeled using mixing of data from a homogeneous cell population with deep clear PMDs (such as IMR90) and a homogeneous cell population classified as PMD- and a high global % methylation. What percentage of cells with PMDs is needed in order to detect PMDs within a population? This is currently unknown. These models could be applied to the T cell differentiation data set shown in the new Supplemental Fig S19, but with multiple comparison lines representing the different percentage of cells within the population containing PMDs.

Additionally, this study could really provide novelty by the analysis of some of the early single cell methylome data sets or by developing new single cell data sets in the T cell differentiation models. The authors argument that the coverage is not sufficient does not appear valid considering that PMDs are large domains that can be called in very low coverage datasets, particularly if using the new method of solo-WCGW method of PMD detection described in Zhou et al.

As the manuscript stands, it is potentially interesting but there are really no new findings from these rudimentary analyses that have not been described previously.

**Reviewer 2**

The authors have answered all my concerns.

Authors Response

Point-by-point responses to the reviewers’ comments:

Reviewer #1: The authors have made a reasonable attempt to respond to reviewer critiques, although mostly with wording changes and written responses rather than new experimental or comprehensive bioinformatic analyses. My prior concerns about lack of novelty of the studies analyses and findings still stand.

Unfortunately for the authors, a new publication on a very comprehensive analysis of PMDs across a larger sampling of WGBS data sets was recently published and is attached. Therefore the author's claim in the abstract "Here, we present a first comprehensive comparative analysis of PMDs across 171 and 24 WGBS methylomes of human and mouse cells, respectively..." is no longer appropriate after the Zhou et al publication.

We agree and adapted the text (p:2, line 6, p:9, line:32, p:14, line:5). However we would like to stress that the analysis of Zhou et al. differs from our analysis as they entirely focus on solo-WCGW positions and many of their datasets are non-WGBS data such as 450K arrays. Their focus was on defining common PMDs/HMDs i.e. shared across many samples by solo-WCGW method. In this respect our work is much more fine grained using all CpGs allowing us to identify/define cell type-specific PMDs/HMDs (in addition to common PMDs/HMDs) only using WGBS data sets of (mainly) primary cells. If anything, our work therefore complements and extends the analysis.

What could be novel about the current study, if the authors are willing to address the question head on and aggressively is the major unanswered question in the field: is the gain of PMDs with mitotic cell divisions due to demethylation of all the cells or clonal selection of a smaller subset of cells that already contain PMDs. The Zhou et al manuscript, while it did other analyses quite elegantly, was not able to answer this question, leading to the rather squishy wording in the title that PMDs are "linked" to mitotic cell division (rather than caused or associated). The authors attempt to answer my concern about this outstanding question, but the analysis was unsatisfying. While the "epipolymorphism score" appears to be a reasonable approach, the model of a simple bimodal population of completely methylated and completely unmethylated molecules is not what is expected in an actual situation where a cell population containing <1% of cells containing PMDs (so apparently PMD-) expands to 10%, 20%, 50%, etc of the cell population through clonal expansion, resulting in a PMD+ classification of the cell population. These cell population shifts could be bioinformatically modeled using mixing of data from a homogeneous cell population with deep clear PMDs (such as IMR90) and a homogeneous cell population classified as PMD- and a high global % methylation.

We agree that the question how demethylation arises in primary cells is and remains unresolved. This was and is not the main focus of our analysis – it’s a very interesting side aspect for which we provide some hints – and we think we were very careful with our interpretations. The reviewer asks for more knowing that this would require a completely a different set of data and a different experimental setup, e.g. following demethylation over time in various dividing and non-dividing primary (!) cells. In fact we now include one such dataset to approach this question for one example (discussed below Figure2).

First of all we performed the proposed in silico simulation. We produced an artificial liver cell mixture by mixing real primary hepatocyte data (randomly chosen) with HepG2 cell line data (note that HepG2 is overall even more hypomethylated than IMR90) in 10% percent incremental steps. We observe a very small decrease of the epipolymorphism score during the “simulated shifts” not reaching or being close to the theoretical minimum based on a bimodal population (see the figure 1A below). This suggests that either the supposedly homogeneous cell population of HepG2 is not really homogeneous or that the real biological samples are more complex than what we model.

We then examined the changes in CpG patterns in HMDs and PMDs across the simulated samples (see the figure 1B below); we found that the fraction of fully methylated patterns (4 consecutive CpGs in the same read) and the fully unmethylated patterns differ between HepG2 and PHH in PMDs but not in HMDs. Most changes in the simulated cell population are found as a decrease of fully methylated patterns and an increase in the unmethylated patterns. Such a scenario is best explained by lack of maintenance after cell division. In this response letter we added the figure 1 to illustrate the outcome.

To approach the mode of demethylation more realistically we also performed the experiment as outlined in the figure 3 below (added to the supplementary material as Figure S19). T memory cells from three different donors were sorted either as a bulk culture (ex vivo samples) or were expanded in a single-cell format (96 well-plate) following stimulation by TCR. After expansion, single clonal cultures were harvested and treated with bisulfite for RRBS analysis. All three ex vivo and the matching three clones were sequenced. As for the natural isolates we observe a preferential loss of methylation in PMDs (as defined by WGBS in Durek et al. 2016) (Figure 2A below).

We inspected the changes in the CpG patterns (as mentioned before) in the ex-vivo samples and the corresponding clone samples. We found that the mixed patterns’ percentage does not change during the cells expansion while the fully unmethylated patterns’ percentage increases at the expense of the fully methylated one (Figure 2B below). Moreover, between 20-35% of the fully methylated ex vivo sample CpGs loses methylation in the clone. This is in line with our findings above which suggests that a loss of methylation with cell division is more likely than a clonal selection considering that the cell population arises from one single cell per donor and that this effect can be observed in all three replicates. Our observation is in line with a recent paper by Dmitrijeva et al 2018 which suggests that at least for cancer cells “hypomethylation is unlikely to be the result of a population level effect only and the extent of hypomethylation is proportional to the cell division rate of the tissue”. We incorporate our results into the discussion (p:12, line:5-25).

In summary, our new experiment suggests that at least in dividing T-cells demethylation is linked to cell division and is unlikely to be a result of clonal expansion. Note that after cells expansion the cell population is ideally homogeneous. The enzymatic mechanism causing demethylation in PMDs (e.g. lack of maintenance) will need further investigations.

What percentage of cells with PMDs is needed in order to detect PMDs within a population? This is currently unknown. These models could be applied to the T cell differentiation data set shown in the new Supplemental Fig S19, but with multiple comparison lines representing the different percentage of cells within the population containing PMDs.

As suggested we utilized the mixing experiment to evaluate the detection rate of MethylSeekR, with the hope of detecting a clear mixing cutoff (which in the reviewer’s assumption would represent the percentage of cells with PMDs). To this end, we applied MethylSeekR on each mixture and subsequently annotated genome-wide tiled 10kb windows to corresponding states. According to the transition matrix between the two extreme samples, pure HepG2 (PHH_0.0) and pure PHH (PHH_1.0), the most common transitions (excluding same-to-same transitions) are, in order, from PMD to UMR, HMD to PMD and HMD to UMR (see figure 3A below). In principle, this is a more detailed summary of the differences between the HepG2 and PHH segmentation.

A closer look at transitions across simulated samples revealed that already in this limited setup a transition depends on the methylation status of the particular region (compare HMD to PMD and HMD to UMR transitions in figure 3B below).

We see that 20% HepG2 is enough to convert 10% of the windows from HMD to PMD, but still with 90% HepG2 there are still more than 30% HMD windows. The same hold for the other two cases but at different mixing thresholds i) HMD to UMR, close to 50% labeled UMR with 90% HepG2 ii) PMD to UMR, transition starts at 60% HepG2, 50% is converted at 70% HepG2 and with 90% HepG2 ~85% are labeled UMR.

Hence, there is no clear cut-off for detecting PMDs/UMRs, but rather it is dependent on the PMD methylation levels which vary strongly between cell types (see MS figure 2A, middle panel with boxplots representing the methylation levels in PMDs across different cell types). Considering the epipolymorphism results mentioned above (Figure 1), we are of the opinion that we do not have a pure PMD control, neither in HepG2 nor IMR90.

In summary, we find that fixing a number to define the “required percentage of cells with PMD” is problematic since PMD calling is dependent on the neighborhood and the overall levels of methylation in individual cell type. Nevertheless we are confident that a robust PMD calling works across many cell types (Figure 2A in the MS).

Additionally, this study could really provide novelty by the analysis of some of the early single cell methylome data sets or by developing new single cell data sets in the T cell differentiation models. The authors argument that the coverage is not sufficient does not appear valid considering that PMDs are large domains that can be called in very low coverage datasets, particularly if using the new method of solo-WCGW method of PMD detection described in Zhou et al.

We performed the clonal expansion experiment mentioned above.

As the manuscript stands, it is potentially interesting but there are really no new findings from these rudimentary analyses that have not been described previously.

We disagree with this statement for the following reasons:

• Our work is a continuation of our colleagues’ previous findings (which were based on small number of datasets); [Berman et al 2012] (two), [Hovestadt et al 2014] (four) and [Schroeder et al 2011/2013] (three). We have performed, as suggested by the latter, a systematic PMD classification to define cell type specific PMDs by integrating hundreds of WGBS samples. Such a comprehensive catalog of cell type-specific PMDs across hundreds of samples has never been published before, also not in in the publication of Zhou et al.

• Our analysis focused on defining cell type-specific PMDs/HMDs (in addition to common PMDs/HMDs) across hundreds of samples by implementing an easy and straight forward workflow. Such analysis has not been reported yet. The work by Zhou et al focuses on defining common PMDs/HMDs using a local sequence signature.

• With “ChromH3M” we provide an “admittingly simple”, but comprehensive novel method for a meta PMD analysis to define cell type specific PMDs in addition to common PMDs/HMDs. ChromH3M is an easy method to analyze large data sets with hundreds of WGBS samples.

• Our findings show that genome wide epigenetic changes found in cell line models should be interpreted with great care. This point has been appreciated by reviewer#1 in the first revision. “Is the paper of broad interest to others in the field, or of outstanding interest to a broad audience of biologists?

Reviewer #1: Yes: The study of PMDs has been a small but emerging subset of papers, but all biologists should be made more aware of their important signature of chromatin state and tissue identity. Therefore, because of its size, this study could help make PMDs more in the mainstream genomic vernacular.”

• We provide novel insights linking PMD heterochromatic signatures to DNA replication timing. While previous studies showed that PMDs are heterochromatic [Hon et al 2012, Hovestadt et al 2014] and late replicating in S phase [Berman et al 2012] our fine grained analysis extends these findings and reveals distinct heterochromatic PMD signatures associated with early/middle S phase, middle/late S phase and late S/G2 phase.

Reviewer #2: The authors have answered all my concerns.

We are grateful for reviewing our manuscript.

List of changes in supplementary figures/materials

We made the following changes to the additional file 2 – supplemental materials:

• We deleted the section “supplementary methods: Epipolymorphism calculation” and the corresponding figure S19.

• We added two new section “supplementary methods: Clonal cultures of T memory cells” and “CpG patterns analysis” with the corresponding Figure S19.

References

Schroeder, D.I., Lott, P., Korf, I., LaSalle, J.M.: Large-scale methylation domains mark a functional subset of neuronally expressed genes. Genome Research 21(10), 1583–1591 (2011)

Schroeder, D.I., Blair, J.D., Lott, P., Yu, H.O.K., Hong, D., Crary, F., Ashwood, P., Walker, C., Korf, I., Robinson, W.P., et al.: The human placenta methylome. Proceedings of the National Academy of Sciences 110(15), 6037–6042 (2013)

Hovestadt, V., Jones, D.T., Picelli, S., Wang, W., Kool, M., Northcott, P.A., Sultan, M., Stachurski, K., Ryzhova, M., Warnatz, H.-J., et al.: Decoding the regulatory landscape of medulloblastoma using dna methylation sequencing. Nature 510(7506), 537–541 (2014)

Berman, B.P., Weisenberger, D.J., Aman, J.F., Hinoue, T., Ramjan, Z., Liu, Y., Noushmehr, H., Lange, C.P., van Dijk, C.M., Tollenaar, R.A., et al.: Regions of focal dna hypermethylation and long-range hypomethylation in colorectal cancer coincide with nuclear lamina-associated domains. Nature Genetics 44(1), 40–46 (2012)

Zhou, Wanding, Huy Q. Dinh, Zachary Ramjan, Daniel J. Weisenberger, Charles M. Nicolet, Hui Shen, Peter W. Laird, and Benjamin P. Berman. "DNA methylation loss in late-replicating domains is linked to mitotic cell division." Nature genetics 50, no. 4 (2018): 591.

Dmitrijeva, Marija, Stephan Ossowski, Luis Serrano, and Martin H. Schaefer. "Tissue-specific DNA methylation loss during ageing and carcinogenesis is linked to chromosome structure, replication timing and cell division rates." Nucleic Acids Research (2018).

Hon, G.C., Hawkins, R.D., Caballero, O.L., Lo, C., Lister, R., Pelizzola, M., Valsesia, A., Ye, Z., Kuan, S., Edsall, L.E., et al.: Global dna hypomethylation coupled to repressive chromatin domain formation and gene silencing in breast cancer. Genome Research 22(2), 246–258 (2012)

Durek, Pawel, et al. "Epigenomic profiling of human CD4+ T cells supports a linear differentiation

model and highlights molecular regulators of memory development." Immunity 45.5 (2016): 1148

1161.
